# Aerobic exercise and a BDNF-mimetic therapy rescue learning and memory in a mouse model of Down syndrome

**DOI:** 10.1038/s41598-017-17201-8

**Published:** 2017-12-04

**Authors:** Martina Parrini, Diego Ghezzi, Gabriele Deidda, Lucian Medrihan, Enrico Castroflorio, Micol Alberti, Pietro Baldelli, Laura Cancedda, Andrea Contestabile

**Affiliations:** 1 Department of Neuroscience and Brain Technologies, Istituto Italiano di Tecnologia, Genova, Italy; 2 Center for Synaptic Neuroscience, Istituto Italiano di Tecnologia, Genova, Italy; 3 Department of Experimental Medicine, University of Genova, Genova, Italy; 4 Present Address: Medtronic Chair in Neuroengineering, Center for Neuroprosthetics, Institute of Bioengineering, School of Engineering, École Polytechnique Fédérale de Lausanne, Lausanne, Switzerland; 5 Present Address: Laboratory of Molecular and Cellular Neuroscience, Rockefeller University, New York, NY USA; 6 Present Address: Laboratory of Neurophysiology, Department of Physiology and Biochemistry, University of Malta, Msida, Malta

## Abstract

Down syndrome (DS) is caused by the triplication of human chromosome 21 and represents the most frequent genetic cause of intellectual disability. The trisomic Ts65Dn mouse model of DS shows synaptic deficits and reproduces the essential cognitive disabilities of the human syndrome. Aerobic exercise improved various neurophysiological dysfunctions in Ts65Dn mice, including hippocampal synaptic deficits, by promoting synaptogenesis and neurotransmission at glutamatergic terminals. Most importantly, the same intervention also prompted the recovery of hippocampal adult neurogenesis and synaptic plasticity and restored cognitive performance in trisomic mice. Additionally, the expression of brain-derived neurotrophic factor (BDNF) was markedly decreased in the hippocampus of patients with DS. Since the positive effect of exercise was paralleled by increased BDNF expression in trisomic mice, we investigated the effectiveness of a BDNF-mimetic treatment with 7,8-dihydroxyflavone at alleviating intellectual disabilities in the DS model. Pharmacological stimulation of BDNF signaling rescued synaptic plasticity and memory deficits in Ts65Dn mice. Based on our findings, Ts65Dn mice benefit from interventions aimed at promoting brain plasticity, and we provide evidence that BDNF signaling represents a potentially new pharmacological target for treatments aimed at rescuing cognitive disabilities in patients with DS.

## Introduction

Down syndrome (DS) is the main cause of genetically defined intellectual disability, with an estimated frequency of 1 in every 1000 live births^1^. Intellectual disabilities represent the major hallmark of DS and are characterized by a low Intelligence Quotient (IQ), as well as learning and memory deficits in different cognitive domains^1–4^. The Ts65Dn mouse is the best characterized and most widely used animal model of DS. It carries a freely segregating extra chromosome bearing the triplication of the distal segment of mouse chromosome 16, which is syntenic to part of the long arm of human chromosome 21^5^. The Ts65Dn mouse recapitulates many aspects of DS, including cognitive impairment and altered hippocampus-dependent memory function^5–7^. Although the Ts65Dn model presents some genetic limitations due to partial triplication of human chromosome 21 (HSA21) orthologous genes and the concurrent triplication of some non-HSA21 genes^8^, it recapitulates many of the phenotypic features of the human syndrome and is currently the only mouse model used for preclinical identification of pharmacological interventions targeting cognitive impairments in DS^9^. A number of mechanisms underlying these cognitive hallmarks have been identified in Ts65Dn mice, including an imbalance between excitatory and inhibitory neurotransmission^10–19^, impairment of hippocampal synaptic plasticity^6,11,20^ and a decrease in adult neurogenesis in the hippocampal dentate gyrus (DG)^6,21^.

According to previous studies of both rodents and humans, aerobic exercise induces a wide range of beneficial effects on brain neurophysiology, including enhanced expression of neurotrophins, increased hippocampal volume and improved cognitive performance^22–39^. The beneficial effects of aerobic exercise have been associated with the upregulation of BDNF expression, increased adult neurogenesis in the hippocampal DG and potentiation of synaptic plasticity^23–25,29–33,40–48^. Indeed, among the many molecular players involved in the positive effect of exercise, BDNF signaling through its tropomyosin-receptor-kinase B (TrkB) receptor is known to play a pivotal role in both enhanced brain plasticity and cognition^47,49–52^. Moreover, many studies have highlighted BDNF/TrkB signaling as a main regulator of long-term potentiation (LTP, a well characterized model of synaptic plasticity) at hippocampal Schaffer collateral-CA1 synapses^53–55^, hippocampus-dependent cognition and learning^56–62^ and the maturation and integration of DG newborn neurons into the hippocampal circuit^63,64^. Interestingly, different manipulations that are able to rescue learning deficits in Ts65Dn mice are associated with increased BDNF expression^17,65–67^, indicating a possible common mechanism of action through the modulation of the neurotrophin system.

Here, we report a detailed biochemical, structural, electrophysiological and behavioral assessment of the positive effect of aerobic exercise on the corresponding deficits observed in trisomic animals, indicating that exercise is a valuable tool to promote brain plasticity and possibly represents a complementary therapeutic approach to pharmacologic interventions in patients with DS. Interestingly, BDNF expression was substantially decreased in the hippocampus of patients with DS, and exercise increased BDNF expression in trisomic animals. Therefore, we investigated the use of a possible pharmacological approach intended to promote BDNF signaling in patients with DS and provide evidence that the treatment of Ts65Dn mice with the BDNF-mimetic drug 7,8-dihydroxyflavone (DHF), completely rescued learning and memory impairments. Thus, we identified a new pharmacological target to treat DS-associated cognitive impairment.

## Results

**Figure 1:**
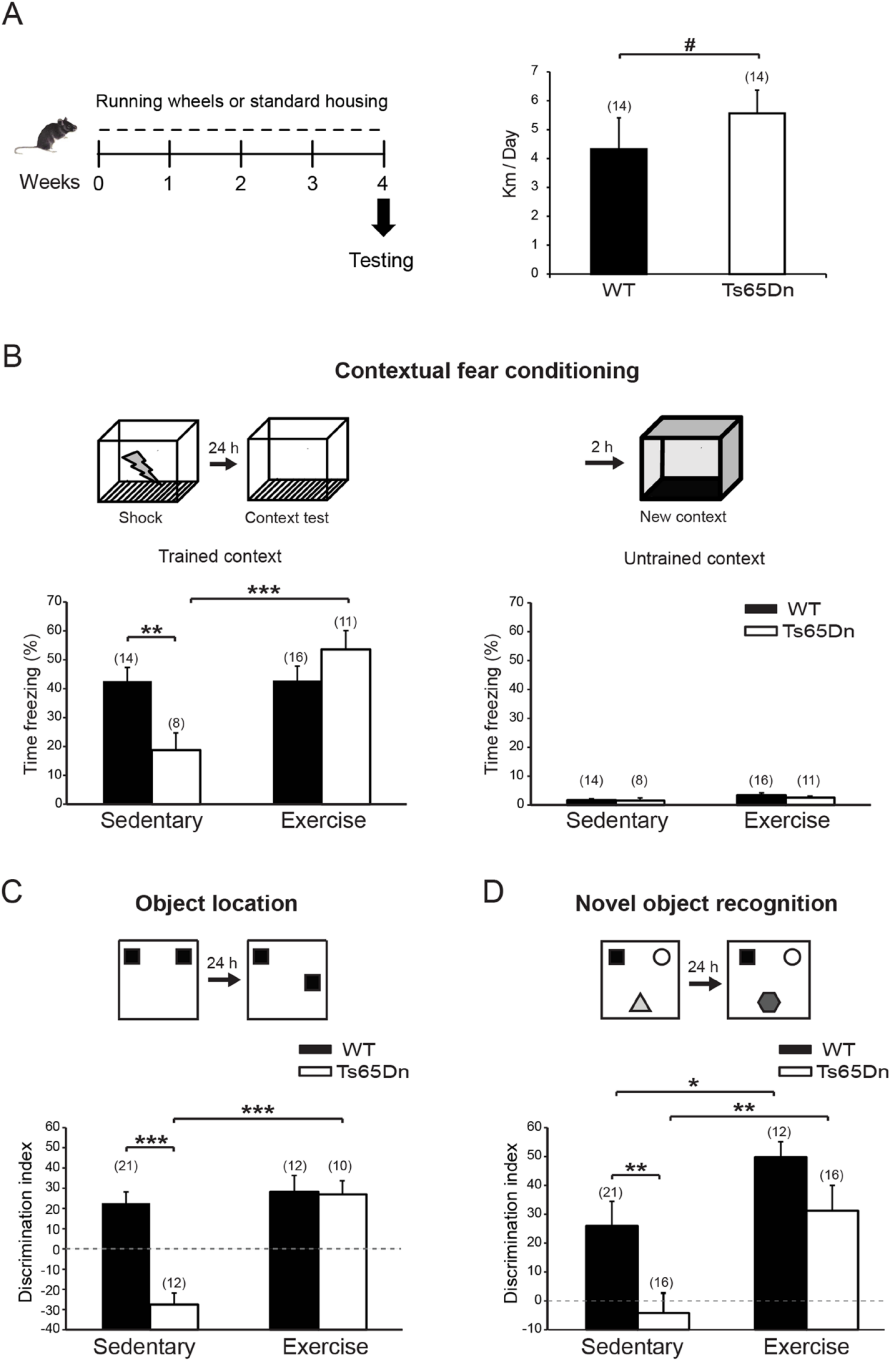
Physical exercise improved cognitive deficits in Ts65Dn mice. (**A**) *Left*, schematic representation of the experimental protocol. *Right*, average daily distance travelled by WT and Ts65Dn mice in the running group. ^#^ *P* = 0.033 Mann-Whitney Test. (**B**) *Top*, schematic representation of the contextual fear conditioning test. *Left*, sedentary Ts65Dn mice showed a strong reduction in freezing response in the context test, indicative of impaired associative learning. Physical exercise completely restored contextual learning in trisomic mice. Two-way ANOVA: genotype [*F*_1,45_ = 1.353, *P* = 0.251]; treatment [*F*_1,45_ = 9.512, *P* = 0.003]; genotype x treatment [*F*_1,45_ = 9.322, *P* = 0.004]. *Right*, both WT and Ts65Dn mice showed negligible freezing response when exposed to a new context. (**C**) *Top*, schematic representation of the object location (OL) test. *Bottom*, four weeks of physical exercise rescued spatial memory in Ts65Dn mice in the OL test. Two-way ANOVA: genotype [*F*_1,51_ = 14.187, *P* < 0.001]; treatment [*F*_1,51_ = 19.382, *P* < 0.001]; genotype x treatment [*F*_1,51_ = 12.787, *P* < 0.001]. (**D**) *Top*, schematic representation of the novel object recognition (NOR) test. *Bottom*, four weeks of physical exercise rescued novelty discrimination deficits in Ts65Dn mice in the NOR test. Two-way ANOVA: genotype [*F*_1,61_ = 8.856; *P* = 0.004]; treatment [*F*_1,61_ = 13.114; *P* < 0.001]; genotype x treatment [*F*_1,61_ = 0.503; *P* = 0.481]. For all panels the number in parenthesis indicates the number of animals tested for each experimental group. *P < 0.05, **P < 0.01, ***P < 0.001, Tukey *post hoc* test following two-way ANOVA.

In this study, we aimed to investigate the effects of strategies that stimulate brain neuroplasticity mechanisms on the rescue of learning and memory deficits observed in the Ts65Dn mouse model of DS. To accomplish this goal, we first took advantage of voluntary exercise, a paradigm known to promote plastic changes and exert beneficial effects on several neurochemical and functional aspects of both the human and rodent brains^22–24^. We hypothesized that aerobic exercise stimulates different neurophysiological processes in the trisomic brain, including adult neurogenesis, synaptogenesis, synaptic plasticity and neurotrophin expression. We provided adult Ts65Dn male mice and wild-type (WT) littermates (4-5 month-old) with free access to running wheels for 4 weeks to assess the effects of physical exercise on these different processes and on cognitive functions in the mouse model of DS (Fig. 1A). Control (sedentary) mice were housed in standard cages without running wheels. Both WT and Ts65Dn mice in the running group (exercise) showed strong running activity that was slightly, but significantly, higher for trisomic animals (WT: 4.33 ± 1.08 Km/day, n = 14; Ts65Dn: 5.56 ± 0.80 Km/day, Fig. 1A).

### Aerobic exercise rescued learning and memory deficits in Ts65Dn mice

Based on accumulating evidence, physical exercise induces specific changes in neural function and enhances learning and memory, particularly hippocampus-dependent behaviors. According to previous behavioral studies, Ts65Dn mice exhibit learning and memory deficits in several behavioral tasks^5–7,10^. We tested Ts65Dn mice in a battery of behavioral tests to evaluate the long-term effects of aerobic exercise on different cognitive domains. Importantly, the exercise paradigm did not cause a general enhancement of motor performance (Supplementary Fig. 1) that could interfere with the evaluation of cognitive functions. We first used the contextual fear conditioning (CFC) test to evaluate associative memory. Consistent with the results from previous studies^6,7^, sedentary Ts65Dn mice showed a strong reduction in the freezing response upon re-exposure to the adverse contest 24 hours after conditioning, indicating an impairment in associative learning in this test. Interestingly, exercise completely restored associative learning in Ts65Dn mice (Fig. 1B), without inducing changes in non-associative freezing (*i*.*e*., exposure to a new context) or altering sensitivity to the shock (Supplementary Fig. 2A).

Next, we evaluated the effect of exercise on spatial memory with the object location (OL) test. In this test, mice must discriminate the new location of a familiar object in relation to the available spatial information. Sedentary Ts65Dn mice were unable to identify the new location after a retention period of 24 hours. However, after 4 weeks of exercise, the performance of Ts65Dn mice was indistinguishable from WT mice, indicating the complete recovery of spatial memory in trisomic animals (Fig. 1C). Next, we used the novel object recognition (NOR) test to evaluate long-term discriminative memory. As expected, sedentary Ts65Dn mice were unable to discriminate the new object, but performed as well as sedentary WT mice following the exercise paradigm (Fig. 1D). Notably, and consistent with other reports, exercise enhanced novel object recognition memory in WT mice^68,69^. The effect of exercise on Ts65Dn mice in both OL and NOR tests was independent of the preference for a different object (Supplementary Fig. 2B,C).

### Aerobic exercise restores functional hippocampal adult neurogenesis in Ts65Dn mice

**Figure 2:**
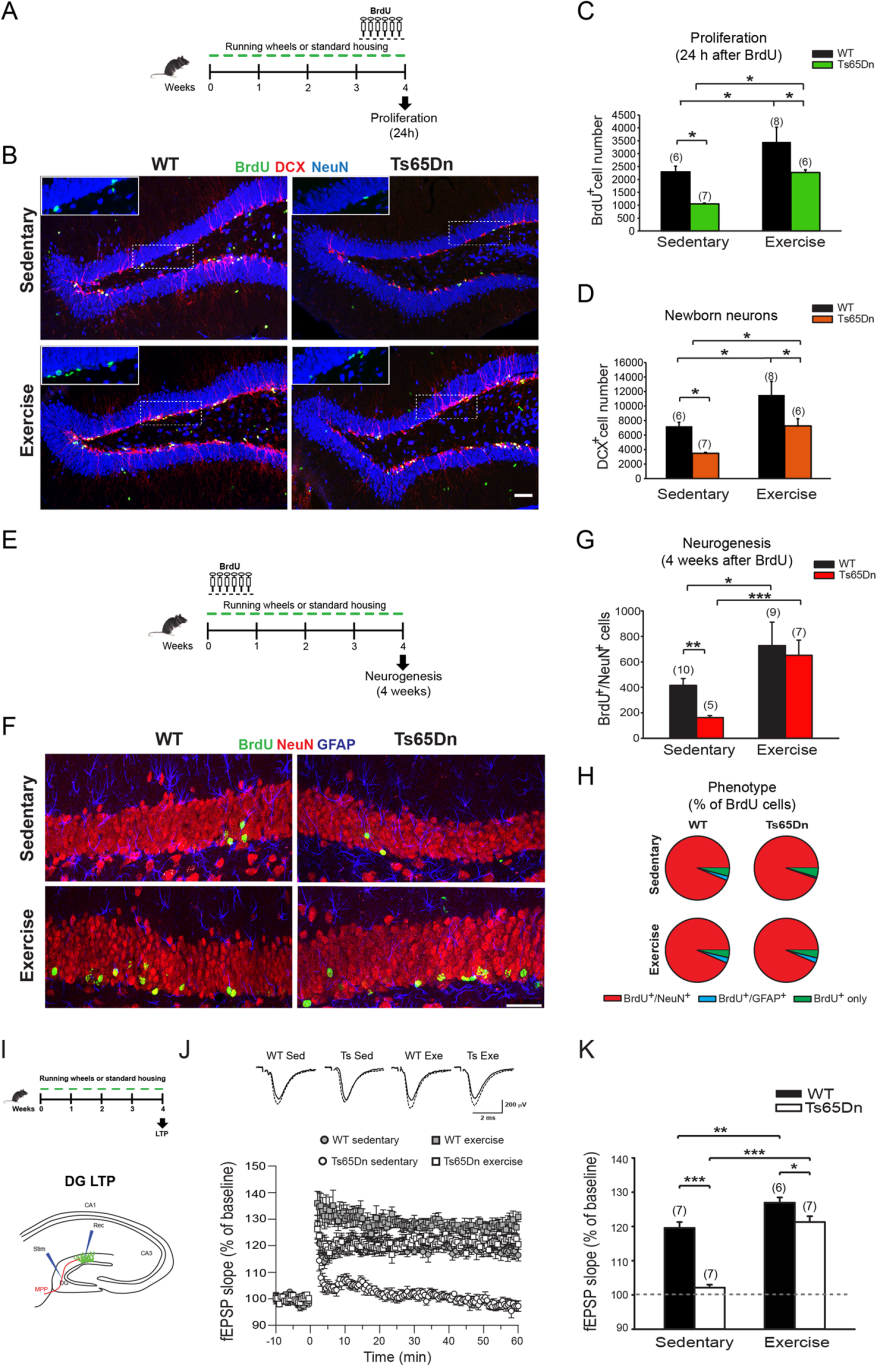
Voluntary exercise restored functional adult neurogenesis in the DG of Ts65Dn mice. (**A**) Schematic representation of the experimental protocol and BrdU administration to label proliferating cells. (**B**) Representative confocal z-stack projection images showing immunostaining for BrdU (green), DCX (red) and the pan-neuronal marker NeuN (blue). Scale bar: 50 µm. The insert on the upper-left corner of each image shows a detail of BrdU staining in the area indicated by the dashed rectangle. (**C**) Quantitative analyses of BrdU^+^ cells found 24 h post BrdU administration in sedentary or running Ts65Dn and WT mice. Proliferation of neural precursor cells was fully rescued by exercise in Ts65Dn mice. Two-way ANOVA: genotype [*F*_1,23_ = 10.967, *P* = 0.003]; treatment [*F*_1,23_ = 11.391, *P* = 0.003]; genotype x treatment [*F*_1,23_ = 0.0000291, *P* = 0.996]. (**D**) Quantitative analysis of DCX^+^ cells in sedentary or running Ts65Dn and WT mice. Numbers of DCX^+^ immature newborn neurons were totally restored in Ts65Dn mice by running. Two-way ANOVA: genotype [*F*_1,23_ = 10.244, *P* = 0.004]; treatment [*F*_1,23_ = 10.480, *P* = 0.004]; genotype x treatment [*F*_1,23_ = 0.0455, *P* = 0.833]. (**E**) Schematic representation of the experimental protocol for BrdU pulse-chase experiments. (**F**) Representative confocal z-stack projection images showing immunostaining for BrdU (green), NeuN (red) and GFAP (blue) on brain slices from mice sacrificed 4 weeks after BrdU administration. Scale bar: 50 µm. (**G**) The number of new neurons (BrdU^+^/NeuN^+^) found 4 weeks after BrdU administration was rescued by exercise in Ts65Dn mice. Two-way ANOVA: genotype [*F*_1,27_ = 5.123, *P* = 0.032]; treatment [*F*_1,27_ = 22.833, *P* < 0.001]; genotype x treatment [*F*_1,27_ = 4.871, *P* = 0.036]. (**H**) Percentage distribution of BrdU^+^/NeuN^+^, BrdU^+^/GFAP^+^, and BrdU^+^ only-labeled cells found 4 weeks after BrdU administration. No statistical difference was found between the experimental groups. (**I**) Schematic representation of the experimental protocol and positioning of stimulating and recording electrodes in hippocampal circuit for DG-LTP. (**J**) Average time course of the increase in the slope of field excitatory postsynaptic potentials (fEPSP) elicited in the DG medial molecular layer after stimulation of the MPP in hippocampal slices obtained from Ts65Dn and WT mice. (**K**) Quantification of LTP elicited in the DG of WT and Ts65Dn mice. Two-way ANOVA: genotype [*F*_1,23_ = 60.530, *P* < 0.001]; treatment [*F*_1,23_ = 79.844, *P* < 0.001], genotype x treatment [*F*_1,23_ = 15.859, *P* < 0.001]. Number in parenthesis indicates the number of samples analyzed for each experimental group. *P < 0.05, **P < 0.01, ***P < 0.001, Tukey *post hoc* test following two-way ANOVA.

Adult neurogenesis in the DG is involved in hippocampus-dependent memory functions^70^ and is known to be regulated by a variety of factors, such as age, stress, exercise, environment, learning, and seizures^46,48,70–72^. In particular, voluntary wheel-running robustly enhances cell proliferation and the number of newly generated neurons in the dentate subgranular zone (SGZ) of the hippocampus^43,46,48^. Since hippocampal cell proliferation and neurogenesis are impaired in adult Ts65Dn mice^6,21^, we thus assessed whether voluntary aerobic exercise positively impacted adult hippocampal neurogenesis in Ts65Dn mice. We first evaluated the proliferation of neural progenitor cells (NPCs) in the DG by labeling dividing cells with the thymidine analog 5-bromo-2-deoxyuridine (BrdU) during the last week of wheel running exercise (Fig. 2A). As expected, the number of BrdU-positive cells (BrdU^+^) cells was substantially decreased in sedentary Ts65Dn mice compared to WT mice, consistent with a proliferation deficit in trisomic adult NPCs^6,21^. However, aerobic exercise induced a 2-fold increase in the number of BrdU^+^ cells in the DG of Ts65Dn mice and also substantially increased proliferation in WT littermates (+50%) (Fig. 2B,C). Accordingly, an immunohistochemistry analysis of the population of adult-generated newborn neurons for the early neuronal marker doublecortin (DCX)^73^ showed a significant reduction in the number of DCX^+^ newborn neurons in the DG of sedentary Ts65Dn mice that was completely restored to levels comparable with sedentary WT littermates following exercise (Fig. 2B–D). In addition, quantification of the long-term survival of newly generated cells and their differentiation toward the neuronal lineage using BrdU pulse-chase experiments (Fig. 2E) revealed that the number of BrdU^+^ cells that were double-labeled with the mature neuronal marker NeuN (BrdU^+^/NeuN^+^) 4 weeks after BrdU administration was significantly reduced in sedentary Ts65Dn mice. However, the number of BrdU^+^/NeuN^+^ cells increased in both WT and Ts65Dn mice following running training (Fig. 2F,G). Moreover, the phenotypic analysis revealed that the percentage of BrdU^+^/NeuN^+^ cells among the total number of BrdU^+^ cells was similar in all groups, indicating that the commitment toward the neuronal lineage was virtually unaffected (Fig. 2H). Based on these data, aerobic exercise efficiently restored adult neurogenesis defects in Ts65Dn mice essentially by rescuing defective NPC proliferation.

We next examined a form of long-term potentiation at medial perforant path (MPP) synapses onto dentate granule neurons (DG-LTP, Fig. 2I), which is known to depend on adult-generated newborn neurons^74–77^, to assess the functional integration of adult-born neurons in Ts65Dn mice following exercise. As shown in our previous study, this *bona fide* form of neurogenesis-dependent synaptic plasticity is absent in Ts65Dn mice due to impaired adult neurogenesis and a decreased supply of newborn neurons^6^. According to previous studies, high frequency stimulation (HFS) of the MPP readily induced DG-LTP in WT slices^6,74–76^, but failed to potentiate slices from Ts65Dn mice (Fig. 2J,K). Physical exercise completely restored DG-LTP in Ts65Dn mice (+19%) to levels similar to their sedentary WT littermates. Physical exercise also slightly, but significantly, increased DG-LTP in WT mice (+7%). Thus, physical exercise rescued functional adult neurogenesis in the DG of Ts65Dn mice.

### Aerobic exercise rescued synaptic deficits in Ts65Dn mice

**Figure 3:**
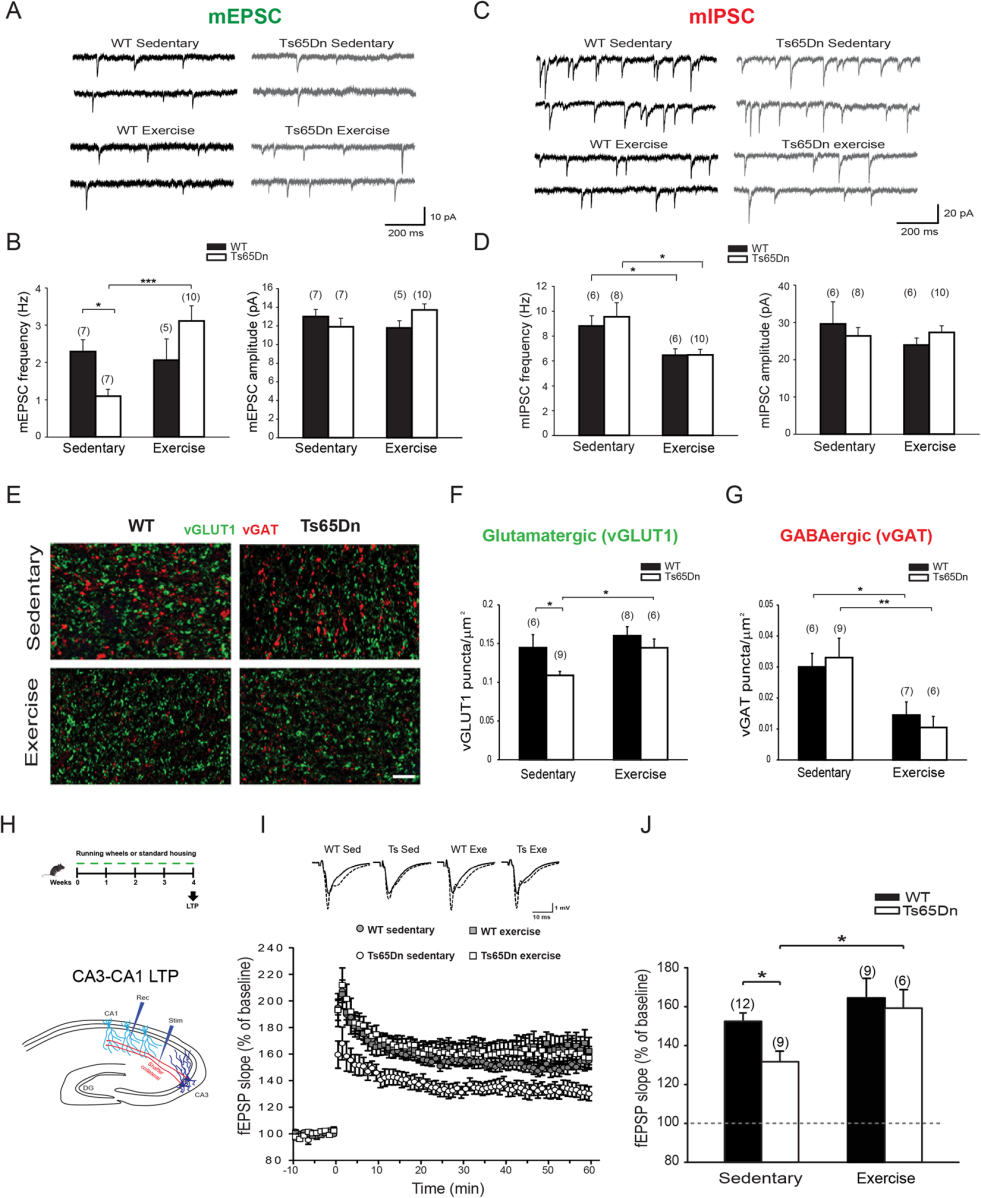
Physical exercises rescues synaptic deficits in Ts65Dn mice. (**A**) Representative traces showing spontaneous miniature excitatory postsynaptic currents (mEPSC) recorded from CA1 pyramidal neurons of WT and Ts65Dn mice in the presence of GABA receptors blockers. (**B**) mEPSC frequency was reduced in sedentary Ts65Dn mice and fully restored by physical exercise. Two-way ANOVA: genotype [*F*_1,25 = _0.0361, *P* = 0.851]; treatment [*F*_1,25 = _5.076, *P* = 0.033]; genotype x treatment [*F*_1,25_ = 8.018, *P* = 0.009]. mEPSCs amplitude was not significantly different across experimental groups. (**C**) Representative traces showing spontaneous miniature inhibitory postsynaptic currents (mIPSC) recorded from CA1 pyramidal neurons of WT and Ts65Dn mice in the presence of glutamate receptors blockers. (**D**) mIPSC frequency was reduced by running in both WT and Ts65Dn mice. Two-way ANOVA: [*F*_1,26_ = 0.0497, *P* = 0.825]; treatment [*F*_1,26_ = 14.628, *P* < 0.001]; genotype x treatment [*F*_1,26_ = 0.0348, *P* = 0.854]. mIPSC amplitude was not significantly different across experimental groups. (**E**) Representative confocal images showing VGAT (red) and VGLUT1 (green) immunostaining. Scale bars: 2 µm. (**F**) The number of VGLUT1-positive glutamatergic synapses was fully rescued by exercise in the CA1 region of Ts65Dn hippocampus. Two-way ANOVA: genotype [*F*_1,25_ = 5.293, *P* = 0.030]; treatment [*F*_1,25_ = 5.239, *P* = 0.031]; genotype x treatment [*F*_1,25_ = 0.806, *P* = 0.378]. (**G**) The number of VGAT-positive GABAergic synapses was decreased by exercise in the CA1 region of both Ts65Dn and WT mice. Two-way ANOVA: genotype [*F*_1,24_ = 1.665, *P* = 0.209]; treatment [*F*_1,24_ = 15.933, *P* < 0.001]; genotype x treatment [*F*_1,24_ = 0.328, *P* = 0.572]. (**H**) Schematic representation of the hippocampal circuit and positioning of the stimulating and recording electrodes for LTP at Shaffer collateral-CA1 synapses (CA3-CA1 LTP). (**I**) Average time course of the increase in the slope of fEPSP elicited in the CA1 Stratum Radiatum by stimulation of the Shaffer collateral in hippocampal slices obtained from Ts65Dn and WT mice. (**J**) Quantification of CA3-CA1 LTP. Two-way ANOVA: genotype [*F*_1,32_ = 3.126, *P* = 0.087]; treatment [*F*_1,32_ = 7.269, *P* = 0.011]; genotype x treatment [*F*_1,32_ = 1.109, *P* = 0.300]. The number in parenthesis indicates the number of sample analyzed for each experimental group. *P < 0.05, **P < 0.01, ***P < 0.001, Tukey *post hoc* test following two-way ANOVA.

We next evaluated possible effects of exercise on non-neurogenic regions of the trisomic brain. Therefore, we first recorded spontaneous miniature excitatory and inhibitory postsynaptic currents (mEPSCs and mIPSCs, respectively) by obtaining whole-cell patch-clamp recordings from hippocampal CA1 pyramidal neurons. Miniature synaptic events (recorded in the presence of the voltage-gated sodium channel blocker TTX to prevent action potential firing) reflect the spontaneous quantal release of neurotransmitters from all presynaptic terminals converging on the recorded neuron. The frequency of these events is proportional to the total number of presynaptic terminals on the patched neuron and the probability of release at each terminal. In sedentary Ts65Dn mice, the mEPSC frequency was significantly reduced, indicating a presynaptic impairment at glutamatergic terminals. Interestingly, the mEPSC frequency was fully restored by exercise in Ts65Dn mice to a level comparable to WT mice (Fig. 3A,B). The change in the frequency was not accompanied by an alteration in the amplitude of these events, suggesting that postsynaptic glutamatergic receptors functioned normally in Ts65Dn mice. In contrast, the mIPSC frequency was similar between sedentary WT and Ts65Dn pyramidal CA1 neurons, but it was significantly reduced by running in both genotypes, without detectable changes in amplitude, consistent with previous reports^78^ (Fig. 3C,D). These electrophysiological results revealed a previously unrecognized synaptic deficit that was specific for glutamatergic synapses in the trisomic CA1 field and was effectively rescued by exercise. In particular, the decrease in mEPSC frequency suggests a decrease in the number of glutamatergic synapses in Ts65Dn hippocampus. We evaluated the density of glutamatergic and GABAergic synapses in the hippocampal CA1 stratum radiatum by immunostaining for the specific presynaptic markers vGLUT1 (vesicular glutamate transporter 1) and vGAT (vesicular GABA transporter), respectively, to elucidate this hypothesis. Consistent with the observed decrease in mEPSC frequency, the density of vGLUT1-positive puncta was decreased in sedentary Ts65Dn mice, indicating a substantial paucity of glutamatergic synapses. Remarkably, aerobic exercise completely restored the density vGLUT1-positive puncta in Ts65Dn mice and significantly decreased the density of vGAT-positive puncta in both WT and Ts65Dn mice (Fig. 3E–G), indicating that exercise extensively regulated synaptogenesis. Moreover, an ultrastructural morphometric analysis using transmission electron microscopy (TEM) showed that the density of total synaptic vesicles (SVs) and the synaptic bouton area were similar in all experimental groups for both asymmetric (glutamatergic) and symmetric (GABAergic) synapses (Supplementary Fig. 3). Conversely, the length of the active zone (AZ) in asymmetric synapses was increased by exercise in both genotypes, whereas the density of docked SVs in symmetric synapses was increased in Ts65Dn mice following exercise. However, these ultrastructural differences were relatively small compared to the changes observed in the density of vGLUT1- and vGAT-positive puncta (Fig. 3E–G).

Since exercise completely restored synaptic deficits in the CA1 region of trisomic animals, we next investigated whether this observation translated to a rescue of the impairment in synaptic plasticity in Ts65Dn mice. We next evaluated LTP induction elicited by theta burst stimulation (TBS) at the Schaffer collateral-CA1 pathway (CA3-CA1 LTP, Fig. 3H) to address this question, and found that potentiation was significantly lower in slices from sedentary Ts65Dn mice than in slices from WT littermates (Fig. 3I,J), similar to a previous report^20^. However, one month of physical exercise increased LTP induction at CA3-CA1 synapses in Ts65Dn mice (+28%) to levels indistinguishable from sedentary WT mice, whereas potentiation was unaltered in WT littermates (*P* = 0.21). Thus, physical exercise indeed effectively restores hippocampal synaptic plasticity in Ts65Dn mice in the CA3-CA1 circuit.

### Aerobic exercise promoted BDNF expression

**Figure 4:**
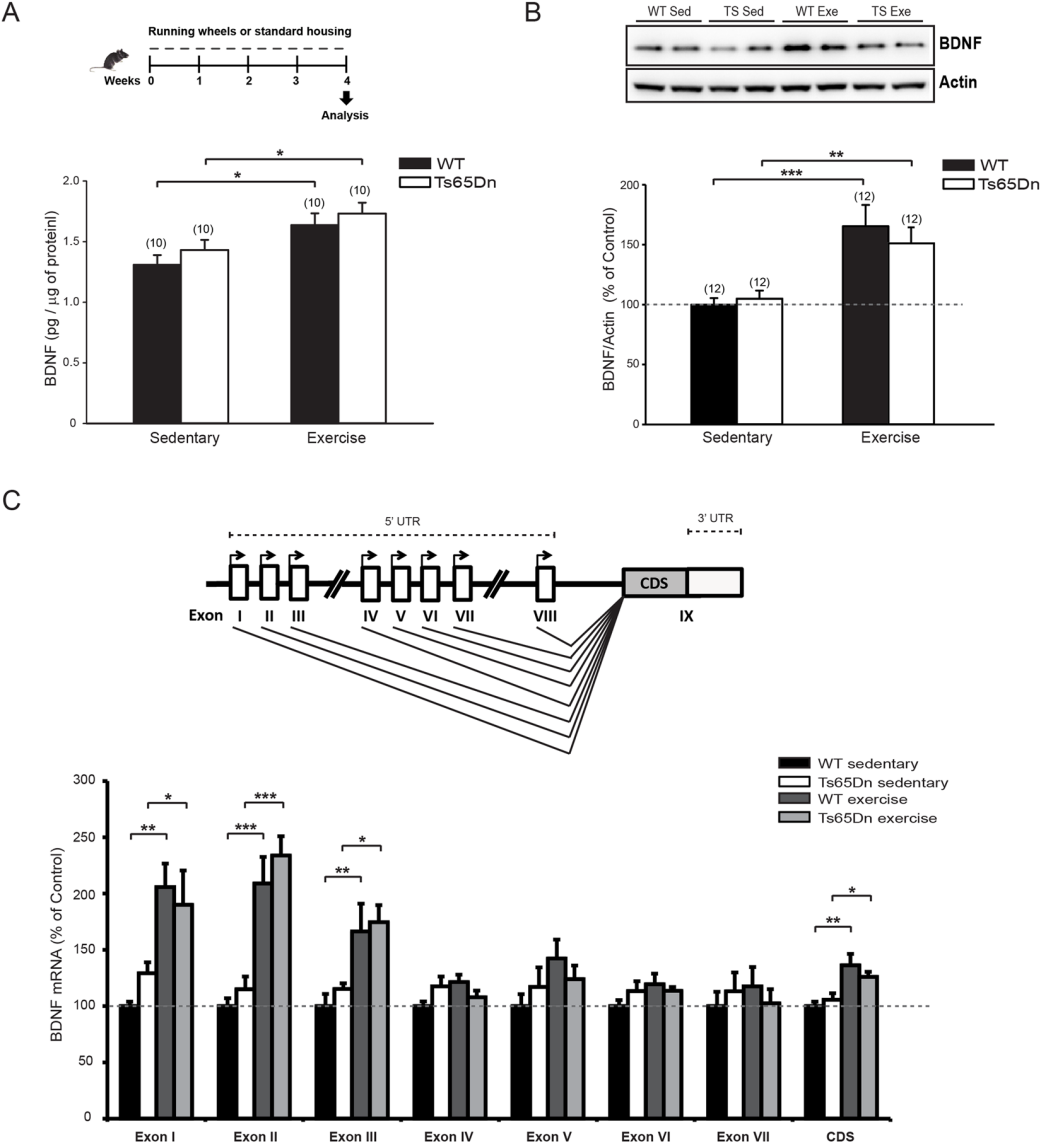
Physical exercise increased BDNF expression in the hippocampus of mice. (**A**) *Top*, schematic representation of the experimental protocol. *Bottom*, quantification of BDNF expression by ELISA immunoassay showed increased expression in the both WT and Ts65Dn running groups. Two-way ANOVA: genotype [F_1,36_ = 1.534, P = 0.224]; treatment [F_1,36_ = 12.862, P < 0.001]; genotype x treatment [F_1,36_ = 0.0224, P = 0.882]. (**B**) *Top*, representative immunoblot for BDNF in protein extracts from hippocampal samples collected from Ts65Dn and WT mice. *Bottom*, quantification of BDNF (expressed as percentage of sedentary WT) showed increased expression following exercise in the both WT and Ts65Dn mice. Actin was used an internal standard. Two-way ANOVA on ranked-transformed data: genotype [*F*_1,44_ = 0.000, *P* = 1.000]; treatment [*F*_1,44_ = 27.322, *P* < 0.001]; genotype x treatment [*F*_1,44_ = 0.547, *P* = 0.464]. The numbers in parenthesis indicates the number of samples for each experimental group. (**C**) *Top*, schematic representation of mouse *Bdnf* gene showing the exon organization of the 5′ and 3′ untranslated regions (UTR; white) and coding sequence (CDS; grey). *Bottom*, RT-qPCR analysis of mouse BDNF mRNA (expressed as percentage of sedentary WT) showed increased expression of transcripts from exons I, II, III and CDS in the hippocampus of exercised mice (n = 5-6 for each experimental group). *P < 0.01, **P < 0.01, ***P < 0.001, Tukey *post hoc* test following two-way ANOVA. Full-length blots are presented in Supplementary Fig. 7.

Aerobic exercise has been shown to increase the expression of brain-derived neurotrophic factor (BDNF) in the brain^23–25,40,41,79^, and exercise-induced upregulation of BDNF has been shown to be necessary for the enhancement of adult neurogenesis and spatial memory^47,49,50^. We therefore analyzed hippocampal BDNF expression in WT and Ts65Dn mice following the exercise paradigm. According to both ELISA and immunoblot quantification, the expression of the BDNF protein was indeed similarly upregulated by exercise in WT and Ts65Dn mice (+50%) (Fig. 4A,B and Supplementary Fig. 4). BDNF mRNA transcripts are characterized by alternative exon usage at the 5′ untranslated region (UTR) and a common coding sequence (CDS). The expression of these alternative BDNF transcripts is controlled by multiple promoters that differentially contribute to activity-dependent BDNF induction^80–82^. An analysis of the expression of different BDNF transcripts by real-time quantitative PCR (RT-qPCR) revealed that the exercise-induced increase in BDNF expression was mainly due to an enhancement of transcription from exons I, II and III in both WT and Ts65Dn mice (Fig. 4C), indicating that the molecular effectors of exercise that are recruited to enhance BDNF expression are substantially spared in trisomy.

### The BDNF-mimetic drug 7,8-dihydroxyflavone rescued synaptic plasticity and cognitive deficits in Ts65Dn mice

**Figure 5:**
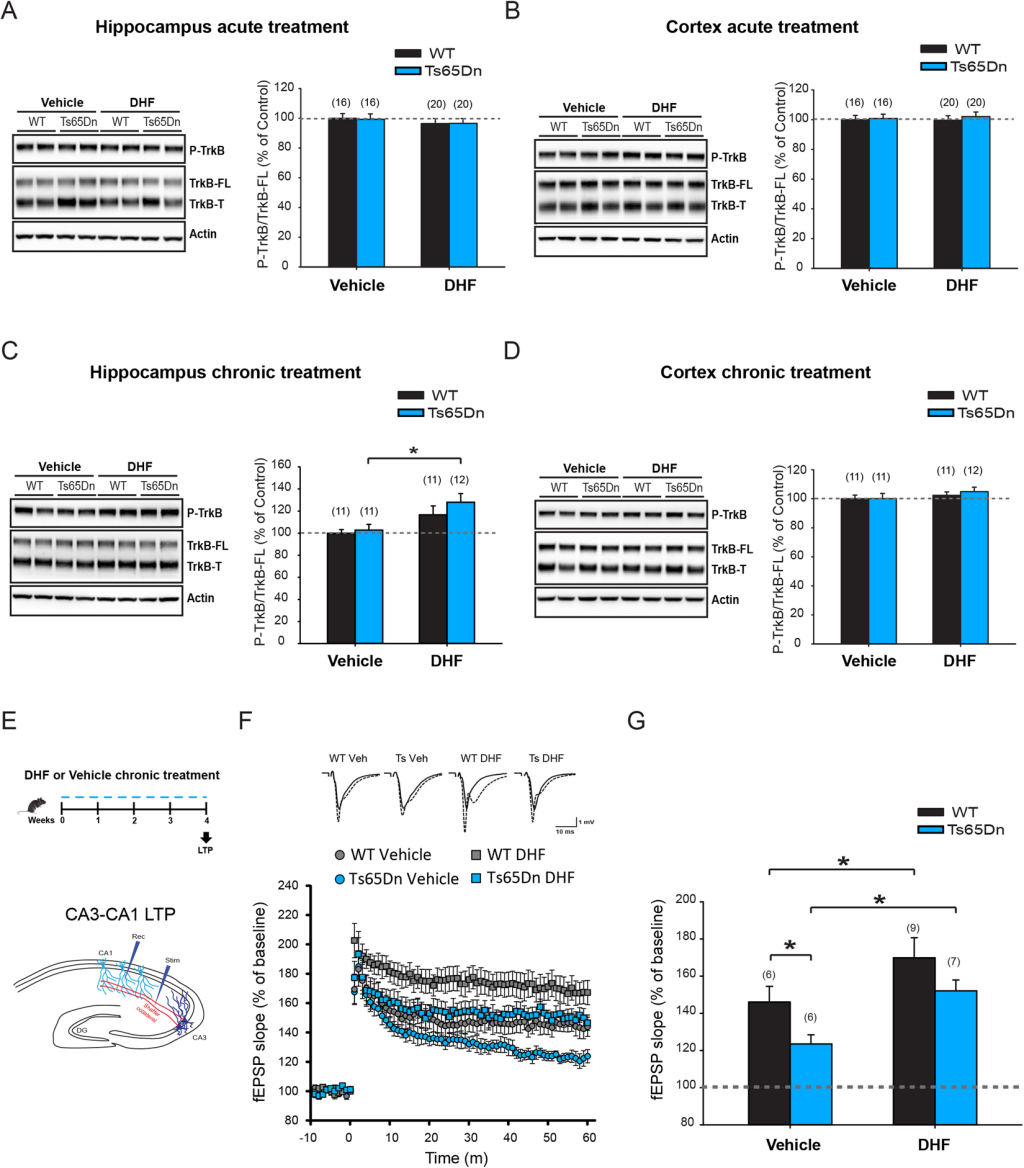
The BDNF-mimetic drug DHF stimulated TrkB signaling and rescued synaptic plasticity deficits in the hippocampus of Ts65Dn mice. (**A**) *Left*, representative immunoblot for phosphorylated (Tyr817) or total TrkB (full-length and truncated isoforms: FL and T, respectively) and Actin in protein extracts from hippocampal samples collected from Ts65Dn and WT mice 1 hour after acute administration of DHF (5 mg/kg) or vehicle. *Right*, quantification of P-TrkB^Tyr817^ (expressed as percentage of vehicle-treated WT) did not show differences after acute DHF administration. Two-way ANOVA on ranked-transformed data: genotype [F_1,68_ = 0.099, P = 0.753]; treatment [F_1,68_ = 1.730, P = 0.193]; genotype x treatment [F_1,68_ = 0.061, P = 0.806]. (**B**) *Left*, representative immunoblot for phosphorylated or total TrkB and Actin in protein extracts from cortical samples collected from Ts65Dn and WT mice 1 hour after acute administration of DHF or vehicle. *Right*, quantification of P-TrkB^Tyr817^ did not show differences after acute DHF administration. Two-way ANOVA: genotype [F_1,68_ = 0.315, P = 0.576]; treatment [F_1,68_ = 0.019, P = 0.892]; genotype x treatment [F_1,68_ = 0.081, P = 0.776]. (**C**) *Left*, representative immunoblot for phosphorylated or total TrkB and Actin in protein extracts from hippocampal samples collected from Ts65Dn and WT mice chronically treated for 4 weeks with DHF or vehicle. *Right*, quantification of P-TrkB^Tyr817^ showed a significant increase in the hippocampus of DHF-treated Ts65Dn mice. Two-way ANOVA on ranked-transformed data: genotype [F_1,41_ = 2.383, P = 0.130]; treatment [F_1,41_ = 9.135, P = 0.004]; genotype x treatment [F_1,41_ = 0.302, P = 0.586]. (**D**) *Left*, representative immunoblot for phosphorylated or total TrkB and Actin in protein extracts from cortical samples collected from Ts65Dn and WT mice chronically treated for 4 weeks with DHF or vehicle. *Right*, quantification of P-TrkB^Tyr817^ did not show differences in the cortex of Ts65Dn and WT mice. Two-way ANOVA: genotype [F_1,41_ = 0.203, P = 0.665]; treatment [F_1,41_ = 1.570, P = 0.217]; genotype x treatment [F_1,41_ = 0.116, P = 0.686]. (**E**) Schematic representation of the experimental protocol and positioning of the stimulating and recording electrodes in hippocampal circuit for LTP at Shaffer collateral-CA1 synapses. (**F**) Average time course of the increase in the slope of fEPSP elicited in the CA1 Stratum Radiatum by stimulation of the Shaffer collateral in hippocampal slices obtained from Ts65Dn and WT mice. (**G**) Quantification of CA3-CA1 LTP. Two-way ANOVA: genotype [F_1,24_ = 8.459, P = 0.008]; treatment [F_1,24_ = 13.530, P = 0.001]; genotype x treatment [F_1,24_ = 0.0593, P = 0.810]. The number in parenthesis indicates the number of sample analyzed for each experimental group. *P < 0.05, Tukey *post hoc* test following two-way ANOVA. Full-length blots are presented in Supplementary Fig. 7.

The data reported above indicate a positive correlation between increased BDNF expression and memory enhancement in Ts65Dn mice. This link is also supported by a large body of evidence showing that many of the positive effects of exercise on the brain are mediated by BDNF^40,41,44,47,49,50^. Therefore, we next investigated whether pharmacological induction of BDNF signaling in trisomic mice would be sufficient to rescue the learning and memory impairment in trisomic animals. Therefore, we took advantage of the recently developed BDNF-mimetic drug 7,8-dihydroxyflavone (DHF), which crosses the brain-blood barrier upon oral administration^83^ and induces BDNF/TrkB signaling^84–95^. Previous reports have shown that acute DHF administration (5 mg/kg body weight) was able to promote TrkB phosphorylation in the brain; yet one study could not detect such effect^84,86,92^. Therefore, we assessed the levels of TrkB phosphorylation after acute DHF administration at the same dose. However, we could not detect any increase in the level of phosphorylated TrkB at Tyr817 (P-TrkB^Tyr817^) in either the hippocampus or cortex of both WT and Ts65Dn mice 1 hour after DHF administration (Fig. 5A,B). Nonetheless, other studies have reported activation of TrkB after chronic DHF treatment^87,90–95^. Accordingly, we found that chronic (4 weeks) administration of DHF significantly increased the level of P-TrkB^Tyr817^ (+26%) in the hippocampus of Ts65Dn mice (Fig. 5C). Notably, DHF-induced TrkB phosphorylation only showed a small increasing trend in the hippocampus of WT animals, but it did not reach statistical significance (*P* = 0.091). Still, in the same group of WT and Ts65Dn animals, we could not detect a significant increase of P-TrkB^Tyr817^ in the cortex (Fig. 5D).

Next, we evaluated whether the chronic DHF treatment rescued hippocampal synaptic plasticity in Ts65Dn mice. The chronic DHF treatment largely phenocopied the effect of exercise on Ts65Dn mice by increasing CA3-CA1 LTP (+23%) to levels similar to vehicle-treated WT animals (Fig. 5E–G). Interestingly, the DHF treatment also increased CA3-CA1 LTP in WT mice (+17%), highlighting the well-known involvement of TrkB signaling in synaptic plasticity^53–55^.

**Figure 6:**
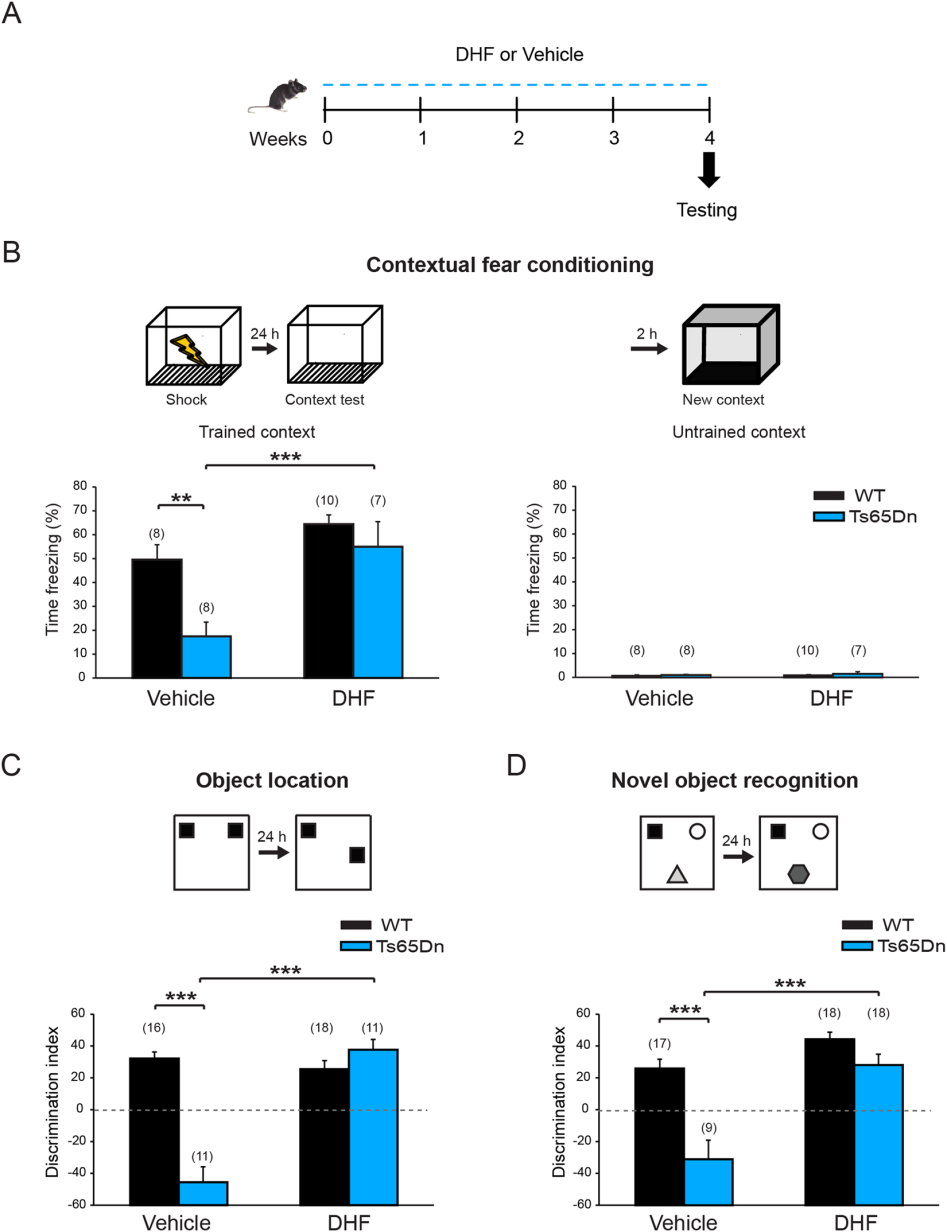
DHF treatment rescued memory deficits in Ts65Dn mice. (**A**) Schematic representation of the experimental protocol. (**B**) *Top*, schematic representation of the contextual fear conditioning test. *Left*, one month of DHF treatment rescued contextual memory in Ts65Dn mice. Two-way ANOVA: genotype [*F*_1,29_ = 9.920, *P* = 0.004]; treatment [*F*_1,29_ = 15.754, *P* < 0.001]; genotype x treatment [*F*_1,29_ = 2.959, *P* = 0.096]. *Right*, both WT and Ts65Dn mice showed negligible freezing response when exposed to a new context. (**C**) Schematic representation of the object location (OL) test. Ts65Dn mice showed an absolute inability to discriminate the new object location after a retention interval of 24 hours. DHF restored spatial memory in Ts65Dn mice. Two-way ANOVA: genotype [*F*_1,52_ = 26.522, *P* < 0.001]; treatment [*F*_1,52_ = 36.311, *P* < 0.001]; genotype x treatment [*F*_1,52_ = 49.978, *P* < 0.001]. (**D**) Schematic representation of the novel object recognition (NOR) test. One month of DHF treatment rescued novelty discrimination deficits in Ts65Dn mice in the NOR test. Two-way ANOVA: genotype [*F*_1,58_ = 27.343; *P* < 0.001]; treatment [*F*_1,58_ = 32.390; *P* < 0.001]; genotype x treatment [*F*_1,58_ = 9.182; *P* = 0.004]. For all panels the number in parenthesis indicates the number of mice tested for each experimental group. *P < 0.05, **P < 0.01, ***P < 0.001, Tukey *post hoc* test following two-way ANOVA.

Furthermore, we evaluated whether the rescue of hippocampal synaptic plasticity induced by the DHF treatment was paralleled by a recovery of cognitive deficits in trisomic animals. Chronic DHF treatment was well tolerated by mice and it did not induce changes in body weight gain or motor activity in either WT or Ts65Dn mice (Supplementary Fig. 5). The DHF treatment indeed rescued associative learning in DS mice in the CFC test, similar to the rescue observed with aerobic exercise (Fig. 6A,B), without inducing changes in non-associative freezing or altering sensitivity to the shock (Supplementary Fig. 6A). Moreover, spatial and discriminative memories were rescued by DHF in the OL and NOR tests (Fig. 6C,D), independent of object preference (Supplementary Fig. 6B,C). In contrast to aerobic exercise and the effects of the DHF treatment on T65Dn mice, DHF did not show a statistically significant effect on WT mice. Based on these data, the DHF treatment rescues different forms of memory in Ts65Dn mice. These data also point to the substantial specificity of the beneficial effect of strategies promoting BDNF/TrkB signaling on DS mice.

### BDNF expression is decreased in the brains of patients with DS

**Figure 7:**
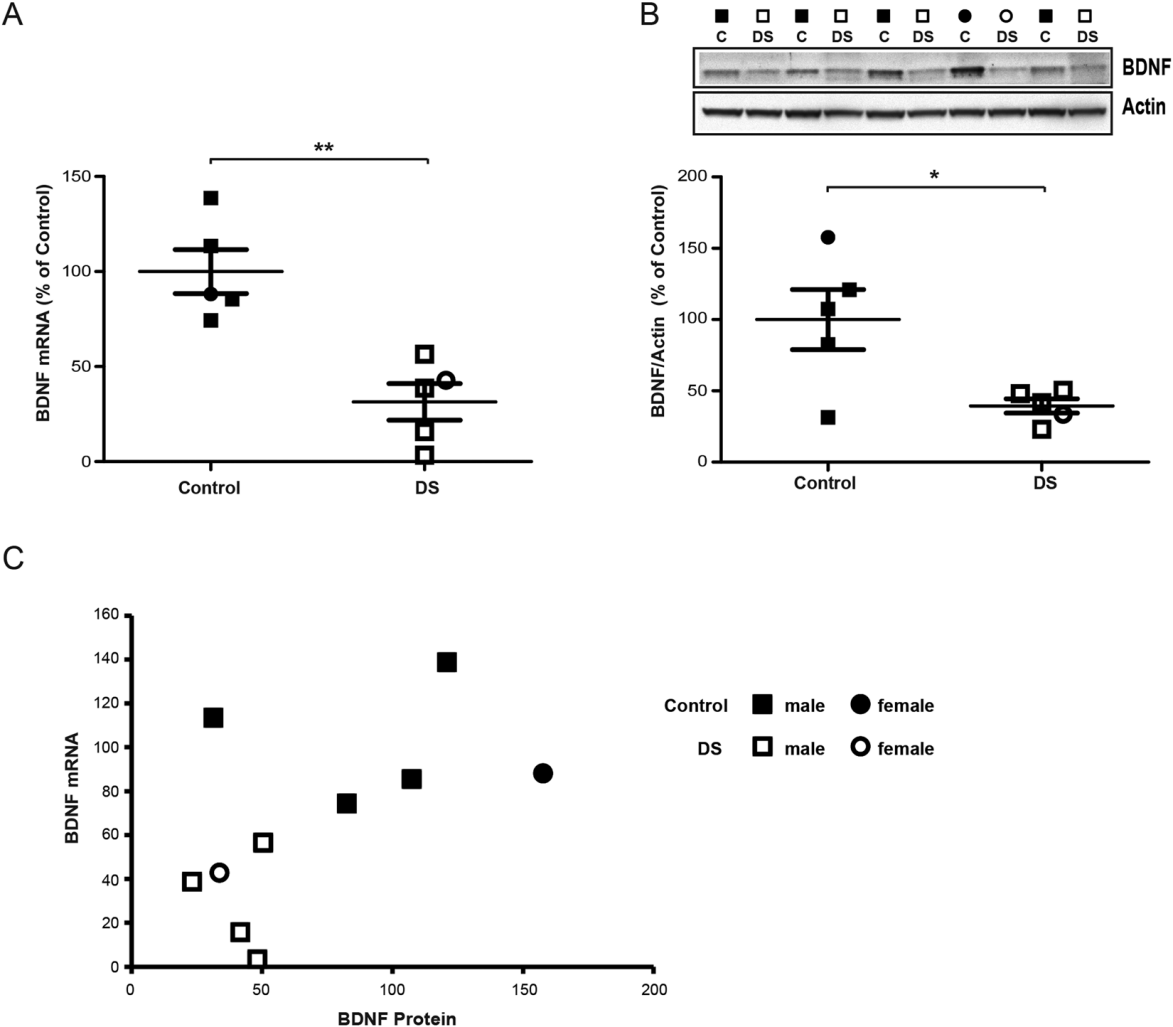
BDNF expression is reduced in the hippocampus DS patients. (**A**) RT-qPCR quantification of BDNF mRNA in the hippocampus of adult DS patients (open symbols: □ males; ○ females) in comparison to age/sex matched non-trisomic controls (solid symbols: ■ males; ● females). Group averages (±SEM) are reported by lines. (**B**) *Top*, representative immunoblot for BDNF in protein extracts from human post-mortem whole hippocampus (gender is indicated above as squares for males and circles for females). *Bottom*, quantification of BDNF (expressed as percentage of controls) showed decreased expression in the hippocampus of adult DS patients in comparison to age/sex matched non-trisomic controls. Actin was used an internal standard. (**C**) Scatterplot representation of BDNF protein and mRNA expression for each individual samples. *P < 0.05, **P < 0.01 unpaired Student’s t-test. Full-length blots are presented in Supplementary Fig. 7.

We next evaluated BDNF expression in the hippocampus of adult patients with DS (13–39 years old) prior to the development of an obvious Alzheimer’s neuropathology to obtain insights into the possible translational applicability of a BDNF-mimetic strategy as a treatment for cognitive impairment. Interestingly, BDNF expression was substantially reduced at both the mRNA and protein levels in the hippocampus of patients with DS compared to age/gender-matched normal controls (Fig. 7A,B), indicating that BDNF/TrkB signaling is likely defective in individuals with DS. As shown it the scatter plot representation of BDNF protein and mRNA expression, although the control and DS groups were clearly separated (Fig. 7C), the correlation between mRNA and protein levels in individual samples was relatively low (Pearson’s correlation coefficients: −0.09 and −0.20 for controls and patients with DS, respectively), indicating a possible dissociation between mRNA and protein levels in post-mortem human samples. However, as baseline BDNF expression is substantially downregulated at both the mRNA and protein levels in the hippocampus of patients with DS, the downstream TrkB signaling pathway is also likely to be compromised in patients with DS, thus highlighting the translational significance of developing a BDNF-mimetic strategy for the treatment of the disease.

## Discussion

Significant cognitive dysfunction is nearly universal across the life span in persons with DS^2,96–101^. Although these cognitive difficulties possibly arise from dysfunction of the hippocampal circuit^2,4,102–105^, we have only recently started to shed light on the neurobiological bases of these abnormalities through the use of mouse models of DS^6,11,13–15,17–20,66,78,106,107^. However, little progress has been achieved in translating these findings into clinically effective treatments to date.

Here, the use of either aerobic exercise or a pharmacological treatment to promote BDNF signaling rescues cognitive symptoms in DS mice. Indeed, according to studies of human subjects and animal models, aerobic physical exercise induces BDNF expression, which, in turn, promotes structural and functional plasticity of neurons, impacting on cognition and resistance to injury and diseases^22–24^.

Using light weight running apparatus with reduced friction that allowed mice to run considerable distances each day, aerobic exercise rescued excitatory synaptic deficits in the hippocampal CA1 region of Ts65Dn mice by promoting functional synaptogenesis of glutamatergic terminals. This effect was paralleled by a substantial recovery of synaptic plasticity in trisomic mice, as revealed by the rescue of defective LTP at Schaffer collateral-CA1 synapses. Accordingly, previous studies have indicated a prominent role for BDNF signaling in regulating synapse formation and LTP induction^108^.

According to several epidemiological studies involving children and adults, physical activity may improve cognitive performance and slow the progression of intellectual decline in aging humans^24–28,32,34–37^. In healthy humans, the levels of aerobic exercise correlate with sustained cerebral blood flow, improved memory performances, increased circulating BDNF levels and increased hippocampal grey matter volume, likely due to the promotion of adult neurogenesis in the DG^29–33,39^. Similarly, in rodents, exercise enhances hippocampus-dependent memory in different behavioral tasks^38^. The beneficial effects of aerobic exercise on rodents have also been associated with an upregulation of the BDNF/TrkB pathway^23–25,40,41^, increased neurogenesis in the hippocampal DG^24,42–46^, and the potentiation of synaptic plasticity^47,48^.

Here, we show an aerobic exercise-induced improvement in learning and memory in different behavioral tasks in DS mice. In particular, we observed a significant improvement in different cognitive domains, including spatial memory (OL test), associative learning (CFC test) and episodic memory (NOR test). Previous studies evaluating the effect of exercise on learning and memory in DS mice have only reported limited efficacy. In the first study, exercise only partially increased spatial acquisition in the Morris water maze (MWM) in relatively old male Ts65Dn mice (aged 10–12 months), but no improvement was detected during the MWM probe trial or in DG adult neurogenesis^109^. A second study did not observe a significant impairment in sedentary female Ts65Dn mice compared to WT mice in the NOR test (but the authors used a different protocol than the protocol we used here). However, a slight increase in preference for the novel object was observed in Ts65Dn females when the mice were allowed access to running wheels beginning at weaning, but not when the exercise paradigm was administered in adulthood (starting from 7 to 17 months of age)^110^. Compared to our strong positive results, one main reason for the previously reported negative outcomes may reside in the low amount of running performed by aged Ts65Dn mice. Similarly, different behavioral tasks used for evaluations of cognitive performances and age- and gender-related differences in the animals may also contributed to the difference between our positive results and previous findings.

Based on our results, the recovery of normal levels of cognitive function was accompanied by both structural and functional rescue of different deficits in the trisomic brains. Consistent with previous studies highlighting the neurogenic effect of aerobic exercise^46,48^, voluntary wheel-running rescued adult neurogenesis in the hippocampal DG of Ts65Dn mice in our study. In particular, wheel-running rescued impaired NPC proliferation and substantially increased the number of newly generated neurons in the DG of Ts65Dn mice. Importantly, the recovery of adult DG neurogenesis was paralleled by the functional integration of the newly generated neurons in the hippocampal circuit, since a *bona fide* form of neurogenesis-dependent dentate LTP^74–77^, which is absent in Ts65Dn mice^6^, was in fact completely rescued by exercise. Consistent with previous studies^46,48,68^, wheel running increased hippocampal adult neurogenesis, DG-LTP and improved cognitive performances in WT mice; these effects where somewhat milder than in trisomic animals. Previous studies have reported a positive correlation between DG adult neurogenesis and cognitive performances in Ts65Dn mice^6,111,112^, highlighting the important role of this form of structural plasticity in the brain on neurophysiology and learning deficits in trisomic animals.

We were also interested in identifying possible effects of exercise that were specific for trisomic animals and occurred in addition to DG adult neurogenesis. Accordingly, we report here for the first time that the frequency of mEPSCs recorded from CA1 pyramidal neurons (*i*.*e*., a non-neurogenic hippocampal region) was decreased in sedentary Ts65Dn mice compared to WT mice and was effectively restored by exercise. Notably, the density of glutamatergic synapses in hippocampal stratum radiatum was also reduced in sedentary Ts65Dn mice and rescued by exercise. These results are consistent with previous immunohistochemical and electron microscopic studies showing a general decrease in the glutamatergic synapse density in the brains of Ts65Dn mice^17,107,113–117^ along with a decrease in the mEPSC frequency in the hippocampal CA3 field^12,17^. Although the effect of exercise on mEPSC frequency and glutamatergic synapse density appeared to be specific for trisomic brains in the present study, exercise also significantly reduced the density of GABAergic synapses and mIPSC frequency in both WT and Ts65Dn mice. We cannot exclude the possibility that the difference in miniature postsynaptic current frequency may at least partially depend on changes in release probability, but the data presented here generally support the conclusion that variations in the number of glutamatergic and GABAergic synapses, rather than alterations in the SV pools^118,119^, are linked to changes in both mEPSC and mIPSC frequencies. Consistent with our data, the density of GABAergic terminals in the brains of Ts65Dn mice was similar to WT mice in previous studies^14,114,115,120,121^. Furthermore, according to previous electrophysiological analyses, the mIPSC frequency, release probability at GABAergic synapses and GABA_A_-mediated transmission were unaffected in the hippocampal CA1 region of Ts65Dn mice^13,78^. However, possible sub-regional differences may exist in the trisomic hippocampus, since other studies have observed an increased GABAergic synapse density in the inner molecular layer of the DG in Ts65Dn mice^19,117^. This finding was supported by the observation that the mIPSC frequency and evoked GABAergic transmission were increased in the DG of Ts65Dn mice^11,14^. The potential occurrence of sub-regional differences in GABAergic innervation was also further highlighted by the observation that the mIPSC frequency was actually decreased in CA3 region of the Ts65Dn hippocampus^12,17^. Most importantly, Ts65Dn synaptic plasticity deficits and cognitive impairments were suggested to arise from an imbalance between excitatory and inhibitory neurotransmission^10–16^. Based on our results, running-mediated recovery of the excitatory/inhibitor balance was paralleled by the selective rescue of LTP at CA3-CA1 synapses in exercised Ts65Dn mice. Consistent with previous studies, we did not observe significant differences in CA3-CA1-LTP between sedentary and running WT animals^48^, although exercise-induced changes in the GABAergic synapse density and mIPSC frequency (but not glutamatergic synapse density and mEPSC frequency) were similar to the changes observed in Ts65Dn littermates. Therefore, the dual effects of physical exercise on both glutamatergic and GABAergic synapses in Ts65Dn mice likely contributes to the observed rescue of synaptic plasticity and cognitive performance.

Similar to aerobic exercise, environmental enrichment (EE) also represents a non-pharmacological method to promote brain plasticity, adult neurogenesis, BDNF expression and learning^71,122,123^. Indeed, previous studies on Ts65Dn mice exposed to EE have shown a substantial recovery of synaptic plasticity and cognitive performances, as well as increased BDNF expression and reduced neurotransmitter release from GABAergic terminals^65,124^. In this regard, enriched cages were also equipped with running wheels in these experiments; therefore, some of the beneficial effects of EE may actually be ascribed to exercise. Interestingly, exercise has recently been shown to be a key factor that mediates increased BDNF expression, adult neurogenesis and enhanced cognitive performances in response to EE^68,79,125^. Our results showing a reduced density of vGAT-positive puncta and mIPSC frequency in both WT and Ts65Dn mice after running clearly suggest that exercise is an active component of the ability of the EE paradigm to modulate GABAergic functions. Interestingly, a study of Ts65Dn mice^126^ has suggested EE as a complementary adjuvant to pharmacological therapy with a polyphenolic green tea extract enriched in epigallocatechin-3-gallate (EGCG). Similarly, a recent clinical trial in patients with DS has introduced a computer-based cognitive training paradigm as an adjuvant to a green tea extract treatment^127^. Some encouraging, although limited, reports have indicated that aerobic exercise may be beneficial in improving executive functions in patients with DS^128–130^. Therefore, a controlled exercise paradigm may also be a valuable complementary intervention to pharmacological therapies for patients with DS. Nevertheless, in our experimental setting, animals ran a considerable distance each day, and Ts65Dn mice ran an even greater distance. The increased activity observed in trisomic animals may likely be attributed to the reported hyperactivity of this mouse model^5^. However, from the clinical perspective, when considering the large distance travelled daily by Ts65Dn mice in our experiments, exercise may not be a practical medical intervention, since it strongly depends on motivation, which may be low in individuals with DS. Thus, a pharmacological approach, such as the one we propose here with DHF, mimicking the molecular changes induced by aerobic exercise may be an alternative and valuable therapeutic intervention.

BDNF has been postulated to act as a key mediator of exercise-associated cognitive improvement, enhanced neurogenesis and synaptic plasticity^23,40,44,47,49,50,68^. Similar to previous reports^17,65–67,131,132^, we confirm here that basal hippocampal BDNF levels are similar in young and middle-aged Ts65Dn mice compared to WT mice. However, BDNF expression was reduced in CA1 hippocampal neurons of elderly Ts65Dn mice^133^, indicating an age-related impairment in BDNF expression in trisomic mice. In this regard, some conflicting results have been reported in the literature, since BDNF expression was reduced in the hippocampus of both young and middle-aged Ts65Dn mice^134–137^. As BDNF expression is influenced by previous experience, gender, circadian rhythms or housing conditions^138–142^, differences in these variables and the analysis method used may explain the reported discrepancies. Additionally, BDNF levels are reduced in the hippocampal DG (but not CA1) and frontal and visual cortices of Ts65Dn mice^65,131^, indicating that regional and sub-regional differences in neurotrophin expression may occur in trisomic animals. On the other hand, BDNF expression was previously reported to be decreased in lymphoblastoid cell lines from individuals with DS^143^. Accordingly, we provide new evidence in this study showing that BDNF levels are substantially reduced in the hippocampus of young adults (mean age 23 years) with DS, indicating that in contrast to animal models, the BDNF deficiency occurs earlier in persons with DS. Therefore, a BDNF-mimetic pharmacological intervention in individuals with DS may represent a strong therapeutic opportunity to rescue cognitive disabilities. Based on the known pivotal effect of BDNF signaling on cognition^108,144^, the observed increase in BDNF expression in Ts65Dn mice compared to the basal level may contribute to the beneficial effect of running we observed in these mice. This result is consistent with other studies showing that the recovery of normal levels of cognitive function in Ts65Dn mice induced by different pharmacological interventions or EE are paralleled by increased BDNF expression^17,65–67^. Nevertheless, the BDNF level *per se* does not completely account for its downstream effects, since the neurotrophin is actively released at synapses and acts at both pre- and post-synaptic sites to regulate several processes through different intracellular signaling cascades^108,145–147^. Indeed, a recent study reported reduced surface binding of BDNF-conjugated Quantum dots (QD-BDNF) to Ts65Dn cortical synaptosomes compared to WT synaptosomes, together with a severely blunted response of the phosphorylation cascade downstream of TrkB upon BDNF application^148^, indicating that synaptic BDNF/TrkB signaling is likely altered in Ts65Dn mice.

Experiments assessing whether strategies that increase BDNF signaling compared to its basal level may be sufficient to rescue the cognitive impairment in Ts65Dn mice are worthwhile in view of the possible translation to patients with DS. We therefore searched for a possible pharmacological treatment that mimicked the effect of physical exercise on potentiating BDNF signaling. We tested the effect of the BDNF-mimetic drug DHF that is orally active, crosses the brain-blood barrier, promotes TrkB phosphorylation and has been proven to be safe and well tolerated in rodents and primates^84–95,149–151^. Moreover, according to the results from *in vivo* pharmacokinetic studies in mice, DHF efficiently penetrates the brain after oral administration (50 mg/Kg), showing a brain to plasma concentration ratio of approximately 1:1 and an elimination half-life of about 2 hours^83^. However, an even longer half-life of 4–8 hours was measured in primate plasma after oral administration (30 mg/Kg)^150^. Although DHF has been shown to activate TrkB in the brain after acute administration^85,86^, we failed to detect any increase in TrkB phosphorylation in both WT and Ts65Dn mice after a single dose. Such discrepancy may at least partially arise from difference in the brain area analyzed in previous studies. However, our results are consistent with at least one report showing increased TrkB phosphorylation only after chronic, but not acute DHF treatment in the hippocampus of the Tg2576 mouse model of Alzheimer’s disease^92^. In keeping with this and other reports^87,90–95^, we found that chronic DHF treatment increased TrkB phosphorylation in the hippocampus of Ts65Dn mice. However, we could not find a statistically significant increase in TrkB phosphorylation in the hippocampus of their WT littermates. This result may indicate increased sensitivity to the drug in DS mice compared to WT, a hypothesis supported by previous observations showing increased TrkB phosphorylation in different mouse brain disease models, but not in the corresponding control animals^88,92,94^. Interestingly, the effect of DHF on TrkB phosphorylation was possibly specific for the hippocampus because the level of P-TrkB^Tyr817^ was similar in the cortex of DHF- and vehicle-treated Ts65Dn mice. This indicates a possible different sensitivity to the drug in the cortex or the occurrence of compensatory and/or desensitization mechanisms.

Chronic oral administration of DHF rescued hippocampal synaptic plasticity and cognitive function in Ts65Dn mice. These results largely mimicked the effect of exercise on Ts65Dn mice. However, the DHF treatment enhanced hippocampal synaptic plasticity in WT to similar levels as trisomic animals, but it was unable to promote learning and memory in WT mice. Therefore, in contrast to physical exercise, the DHF treatment appeared to specifically enhance cognitive functions in trisomic animals, but not in WT littermates. Although we have not investigated this aspect, one possible explanation may be that overstimulation of this pathway in animals with intact BDNF signaling, as presumably found in WT mice, will have little effect on cognition. This possibility is corroborated by previous studies showing a positive effect of DHF on learning and memory in cognitively impaired animals, but not in the corresponding WT^89,152^. Alternatively, aerobic exercise may also recruit other signaling pathways relevant for memory in addition to BDNF in WT animals. However, in trisomic animals, where multiple neuronal and synaptic functions (possibly including neurotrophin signaling) are compromised, the stimulation of BDNF signaling may be sufficient to rescue cognitive functions.

Moreover, the effect of DHF appeared to be consistent with previous studies showing that the administration of Neurotropin^®^ (a drug previously proposed as analgesic for chronic pain) or ciliary neurotrophic factor (CNTF) peptide to increase hippocampal BDNF expression in Ts65Dn mice is associated improved spatial memory^134,137^. Regarding translational opportunities afforded by the present results, physical exercise may represent a valuable complementary therapy aimed at rescuing cognitive disabilities in patients with DS.

Although the care of associated medical issue has largely improved the overall health and life expectancy of individuals with DS, no effective pharmacological treatment is currently available to rescue cognitive deficits. Here, we report pre-clinical data on this widely used mouse model of DS showing that either aerobic physical exercise and/or a DHF pharmacological treatment may represent a promising intervention for alleviating the learning and memory deficits of patients with this syndrome.

## Material and Methods

### Animals and treatment

Ts65Dn colony^5^ was maintained by crossing Ts65Dn female to C57BL/6JEi x C3SnHeSnJ (B6EiC3) F1 males (Jackson Laboratories; Bar Harbor, USA). Animals were genotyped by PCR as previously described^8^. Ts65Dn male mice aged between 16 and 20 weeks were used for experiments and wild-type (WT) littermates were used as controls throughout the study. Ts65Dn and WT littermates were randomly assigned to the running or sedentary groups. Running animals (two mice/cage) were housed for 4 weeks with two light weight and reduced friction running wheels (Med Associated). Sedentary mice were housed in standard cages without running wheels. For the assessment of the mean running distance, a cohort of WT and Ts65Dn mice was individually housed for 4 weeks with one running wheel equipped with a wireless revolution counting system (Med Associated). For neuron birth dating in neurogenesis experiments, mice were administrated with 5-Bromo-2-Deoxyuridine (BrdU; 100 mg/kg body weight) through daily i.p. injections, for 6 consecutive days and perfused for immunohistochemistry 24 hours or 4 weeks after the last injection^6,153^.

For neurogenesis assessment and quantification of synapses density (vGAT/vGLUT1, see below), we used mice after concluding behavioral assessment in the NOR or OL test. Due to the more stressful nature of the CFC test, none of the mice subjected to this test was later used for other experiments. Detailed list of mice used in the study is provided in Supplementary Table 4.

For 7,8-dihydroxyflavone (DHF) treatment, the drug was administered through the drinking water, as previously described^149^. DHF (TCI Europe) stock solution was prepared at 100 mg/ml in dimethylsolfoxide (DMSO) and stored in aliquots at −20 °C. This stock solution was diluted at 80 mg/L in drinking water containing 0.2% (w/v) sucrose^149^. The DHF drinking solution was replaced every 2-3 days and water intake monitored throughout the experiments. The average water intake during the four weeks of treatment was used to calculate the average DHF dose received by mice, which was estimated in 22.16 ± 1.64 mg/Day/Kg body weight. Vehicle-treated mice received drinking water containing 0.08% (v/v) DMSO and 0.2% (w/v) sucrose. Chronic DHF treatment appeared to be well tolerated by mice. Only 1 out of 85 vehicle-treated mice and 1 out of 97 DHF-treated mice died during the study.

For acute drug administration, mice were treated as previously described^84,86,92^ with DHF (i.p., 5 mg/Kg body weight) or the corresponding vehicle (10% DMSO in saline) and sacrificed 1 hour later. To minimize possible circadian effects on gene expression, collection of all samples for biochemical analysis was performed between 9.00 and 12.00 AM. Brains were rapidly dissected in ice-cold PBS and immediately snap-frozen.

### Behavioral testing

Behavioral tests were performed on each animal during the 4^th^ week of treatment as previously described^6,15^. Animal behavior was video-recorded throughout the experimental sessions, and subsequently analyzed by a trained operator blind to the experimental groups. WT and Ts65Dn mice were always evaluated in parallel and with the same time schedule. In order to avoid any confounding effect, tests were administrated only once to individual mice and, various cohorts of mice were used for different behavioral testing. Three days before starting the behavioral experiments mice where handled once a day for 5 min each.

#### Object location test (OL)

This test measures spatial memory by evaluating the ability of mice to recognize the new location of familiar object with respect spatial external cues. The test was conducted in a grey acrylic arena (44 × 44 cm) equipped with a digital video recording system. The test was conducted during 3 consecutive days. On the first day mice were first habituated to the empty arena for 15 minutes. The next day the mice were exposed to 2 identical objects for 15 minutes (acquisition session). Object preference was evaluated during this session. On the third day (trial session) one the object was moved to a novel location, and the mice were allowed to explore the objects for 15 minutes. The object and the arena were cleaned with 70% ethanol after each trial. Exploratory behavior of the objects was defined as direct contact with the object by the animal mouth, nose or paws, or if the animal’s nose was within 1 cm of an object (sniffing); any indirect or accidental contact with the objects was not included in the scoring. A discrimination index was calculated as the percentage of time spent investigating the object in the new location minus the percentage of time spent investigating the object in the old location: Discrimination Index = (New OL Exploration Time/Total Exploration Time * 100) − (Old OL exploration time/Total Exploration Time * 100).

#### Novel object location test (NOR)

This test measures the preference of mice for a novel object versus previously encountered familiar objects. The test was conducted in a grey acrylic arenas (44 × 44 cm) equipped with a digital video recording system as above. The NOR test was conducted during 3 consecutive days, similarly to the OL test. On the first day the mice were habituated to the apparatus by freely exploring the empty arena for 15 minutes. The next day (acquisition session), three different objects were placed into the arena and the mice were allowed to explore for 15 minutes. Object preference was evaluated during these sessions. The objects used during the test were different in shape, color, size and material. On the third day (trial session) one the object used during the acquisition session was replaced by a novel object and the mice were allowed to freely explore the objects for 15 minutes. The objects and the arena were cleaned with 70% ethanol between trials. Exploratory behavior of the object was defined as direct contact with the object by the animal mouth, nose or paws, or if the animal’s nose was within 1 cm of an object (sniffing); any indirect or accidental contact with the objects was not included in the scoring. A discrimination index was calculated as the difference between the percentages of time spent investigating the novel object and the time spent investigating the familiar objects: Discrimination Index = (New Object Exploration Time/Total Exploration Time * 100) − (Familiar Object Exploration Time/Total Exploration Time * 100).

#### Motor activity

The distance traveled by mice in the empty arena during the habituation sessions of the OL and NOR tests was measured with ANY-maze tracking software (Stoelting).

#### Contextual fear conditioning test (CFC)

The fear conditioning system (TSE) consisted of a transparent acrylic conditioning chamber (20 × 10 cm) equipped with a stainless-steel grid floor and a digital video recording system. Mice were held outside the experimental room in their home cages prior to testing and individually transported to the conditioning apparatus in standard cages. The mice were placed in the conditioning chamber and 3 minutes later, received one electric shock (2 s, 0.75 mA constant electric current) through the floor grid. Mice were removed 15 s follow the shock. 24 hours later, the mice were placed in the same chamber for 3 minutes (context test) and after 2 hours moved to a new context (black chamber with plastic gray floor and vanilla odor). The chamber was cleaned with 70% ethanol after each trial. The freezing behavior was scored by a trained operator blinded to the experimental groups.

The NOR and OL test were performed in dim light illumination (12–14 Lux), while CFC was conducted with standard illumination (80 Lux). In behavioral experiments, we adopted the following exclusion criteria independently of genotype or treatment (before blind code break). In the CFC test, we excluded mice showing very high non-associative freezing in the new context: more than 30 seconds freezing during the 3 minutes test (5 out of 86 mice). In the OL and NOR test, we excluded animals showing very low explorative behavior: less than 10 seconds of direct objects exploration during the 15 minutes test (2 out of 113 mice for OL test and 4 out of 131 mice for NOR test).

### Immunohistochemistry

Animals were deeply anesthetized and transcardially perfused with 4% paraformaldehyde in 100 mM phosphate buffer (PB), pH 7.4. Brains were collected, post-fixed for 24 h in the same fixative solution, cryo-preserved in 30% sucrose in PB and stored at −80 °C until use. Immunohistochemistry was performed on 30-μm coronal sections, with the following primary antibodies: rat anti-BrdU (clone BU1/75[ICR1], Abcam, catalog n°: ab6326; 1:200); rabbit anti-DCX (Abcam, catalog n°: ab18723; 1:1000); mouse anti-NeuN (clone A60, Millipore, catalog n°: MAB377; 1:250); rabbit anti-GFAP (Abcam, catalog n°: ab16997; 1:100); rabbit anti-vGAT (SYSY, catalog n°: 131-013; 1:700); guinea pig anti-vGLUT1 (SYSY, catalog n°: 135–304; 1:2500). Fluorophore conjugated (Alexa Fluor 488, Alexa Fluor 568, Alexa Fluor 647), goat secondary antibodies (1:1000; Invitrogen) were used for detection. The sections were counterstained with the nuclear dye Hoechst-33342 (Sigma-Aldrich). For BrdU immunohistochemistry, sections were pretreated with 2 N HCl as previously described^6^.

For the assessment of dentate adult neurogenesis, stereological cell counting was performed in serial coronal sections (180 μm apart) covering the complete rostro-caudal extension of the dentate gyrus as previously described^6^. Fluorescence images were captured with a Nikon A1 confocal scanning microscope equipped with a 40X air objective and a motorized stage. For each section, confocal z-stack images (1 μm z-step size) covering the complete dentate gyrus were acquired and DG reconstructed with NIS Element AR software (Nikon). Immunolabeled cells in the granular cell layer (GCL) and the subgranular zone (SGZ, defined as a 10 μm region below the GCL) were counted on the reconstructed images by an operator blind to the experimental groups according to the optical dissector principle^153^.

For quantitative analyses of vGAT- and vGLUT1-positive puncta density, measurements were performed on images acquired with a SP5 confocal scanning microscope (Leica Microsystems), equipped with a 63X oil-immersion objective. Puncta density was evaluated on serial coronal sections (180 μm apart) covering the complete rostro-caudal extension of the hippocampus. Puncta immunostained for vGAT and vGLUT1 were automatically quantified in random fields of the CA1 stratum Radiatum on single-plain optical section images using the particle analysis plugin available in ImageJ software (http://imagej.nih.gov/ij/).

### Electron Microscopy

Coronal brain slices (30 µm thickness) were post-fixed by immersion in 2% glutaraldehyde in 0,1 M sodium Cacodylate buffer (NaCacodylate, pH 7.4). Following aldehyde fixation, slices were rinsed in 0,1 M NaCacodylate buffer and further treated for 1 hours with 1% OsO4 in 1.5% K_4_Fe(CN)_6_ in 0.1 M NaCacodylate at room temperature, *en bloc* stained with 1% uranyl acetate for 45 minutes, dehydrated through a series of graded ethanol solutions, washed in propylene oxide and flat embedded in epoxy resin (Epon 812, TAAB) between two Aclar sheets. After 48 hours of polymerization at 60 °C, a small region corresponding to the hippocampal CA1 was excised from the blocks of resin and cut with an EM UC6 ultramicrotome (Leica Microsystem). Semi-thin section (0,5 μm thickness) was taken from each brain slice, stained with 1% of toluidine blue and analyzed at low magnification to identify the central region of CA1 stratum Radiatum. Ultrathin sections (70 nm thickness) were collected on 200 mesh copper grids and observed with a JEM-1011 microscope (Jeol) operating at 100 kV and equipped with an ORIUS SC1000 CCD camera (Gatan).

Symmetric (GABAergic) and asymmetric (glutamatergic) synapses were morphologically identified based on the shape of the presynaptic bouton and presence of thick and electron-dense post-synaptic density typical of glutamatergic terminals^154,155^. Synaptic bouton areas, length of the active zone (AZ), total and docked SV densities were calculated using ImageJ software. Total SV density was calculated on the total area of each presynaptic bouton. AZ was morphologically identified as the opposition region between pre- and post- synaptic structures with docked SV. Docked SV density was calculated on the length of the active zone of each presynaptic bouton. Only synapses with at least one docked synaptic vesicles were analyzed.

### Biochemistry

Samples were lysed in ice-cold RIPA buffer (1% NP40, 0.5% Deoxycholic acid, 0.1% SDS, 150 mM NaCl, 1 mM EDTA, 50 mM Tris, pH 7.4) containing 1 mM PMSF, 10 mM NaF, 2 mM sodium orthovanadate and 1% (v/v) protease and phosphatase inhibitor cocktails (Sigma). Samples were clarified through centrifugation at *20*,*000 × g* at 4 °C, and the protein concentration was determined using the BCA kit (Pierce). For immunoblot analysis, equal amounts of proteins were run on 4–12% Bis-Tris NuPAGE (Life Technologies) or Criterion-XT (Bio-Rad) gels and transferred overnight at 4 °C onto nitrocellulose membranes (GE Healthcare). Membranes were probed with rabbit anti-BDNF (Santa Cruz Biotechnology, catalog n°: sc-546; 1:500)^66^, rabbit anti-phospho-TrkB^Tyr817^ (Abcam, catalog n°: ab81288; 1:20000), rabbit anti-TrkB (Millipore, catalog n°: 07-225; 1:2000), and rabbit anti-Actin (Sigma, catalog n°: A2066; 1:10000), followed by HRP-conjugated goat secondary antibodies (Thermo Scientific) or fluorophore-conjugated (Cy3 or Cy5) goat secondary antibodies (GE Healthcare). Chemiluminescence signals were revealed with the SuperSignal West Pico substrate (Pierce) and digitally acquired on a LAS 4000 Mini imaging system (GE Healthcare). Fluorescent signals were acquired with Typhoon fluorescent scanner (GE Healthcare). Band intensities were quantified using ImageQuant software (GE Healthcare). For the determination of phospho-TrkB levels in brain lysates, membranes were first probed with the phospho-specific antibody, stripped and re-probed with a second antibody for total TrkB. The specificity of anti-BDNF antibody was verified by immunoblot analysis of recombinant BDNF (PeproTech) (Supplementary Fig. 4).

BDNF quantification by “sandwich” enzyme-linked immunosorbent assay (ELISA) was performed with BDNF E_max_ ImmunoAssay System (Promega) on 50 μg of hippocampal protein extracts (as above) according to the manufacturer’s instructions. Absorbance at 450 nm was measured with a Victor3 plate reader (PerkinElmer).

### Real Time Quantitative PCR (RT-qPCR)

RT-qPCR was performed as previously described^15,156^. RNA was extracted with QIAzol reagent and purified on RNeasy spin columns (Qiagen), which includes an in-column RNAse-free DNAse digestion for the removal of contaminating genomic DNA. RNA samples were quantified at 260 nm with a ND1000 Nanodrop spectrophotometer (Thermo Scientific). RNA purity was also determined by absorbance at 280 and 230 nm. Reverse transcription was performed according to the manufacturer’s recommendations on 1 µg of RNA with QuantiTect Reverse Transcription Kit (Qiagen). SYBR green RT-qPCR was performed in triplicate with 10 ng of template cDNA using QuantiTect master mix (Qiagen) on a 7900-HT Fast Real-time System (Applied Biosystem), using the following universal conditions: 5 min at 95 °C, 40 cycles of denaturation at 95 °C for 15 sec, and annealing/extension at 60 °C for 30 sec. Product specificity and occurrence of primer dimers were verified by melting curve analysis. Primers were designed with Beacon Designer software (Premier Biosoft) in order to avoid template secondary structure and significant cross homologies regions with other gene by BLAST search. For each target gene, primers were designed to target specific transcript variants annotated in RefSeq database (http://www.ncbi.nlm.nih.gov/refseq). In each experiment no-template controls (NTC) and RT-minus controls were run in parallel to the experimental samples. PCR reaction efficiency for each primer pair was calculated by the standard curve method with serial dilution of cDNA. PCR efficiency calculated for each primer set was used for subsequent analysis. All experimental samples were detected within the linear range of the assay. Gene expression data were normalized by the multiple internal control gene method^157^. To determine an accurate normalization factor for data analysis the expression stability of different control genes was evaluated with GeNorm algorithm^157^ available in qBasePlus software (Biogazelle). The tested control genes were: GAPDH (glyceraldehyde-3-phosphate dehydrogenase), PPIA (peptidylprolyl isomerase A) and ACTB (β-actin). Based on the relative expression stability of the control genes calculated by GeNorm analysis, expression data for the different samples were normalized with GAPDH and PPIA. However we noted that expression stability of the control genes was generally similar, therefore comparable expression results were obtained also by normalization with the other control genes. SYBR green primer sequences are reported in Supplementary Table 1. Calibration curves parameters, PCR reaction efficiency and amplicon information are listed in Supplementary Table 2.

### Human Brain Samples

Hippocampal samples from adult human Down syndrome patients and age/sex-matched non-trisomic controls were obtained from the Brain and Tissue Bank for Developmental Disorders at the University of Maryland, Baltimore (MD). Samples information are described in Supplementary Table 3 and were previously reported^15^. Samples were cryo-pulverized in dry ice (−78 °C) with a stainless steel mortar. Aliquots of pulverized tissue were used for protein and RNA extraction.

### Electrophysiology

#### Field recordings

For CA3-CA1 LTP mice were anesthetized with isoflurane and transcardially perfused with an ice-cold ACSF solution with the following composition (in mM): 120 NaCl, 3.5 KCl, 1.25 NaH_2_PO_4_, 1.3 MgSO_4_, 2.5 CaCl_2_, 26 NaHCO_3_ and 10 glucose (pH 7.4), oxygenated with 95% O_2_ and 5% CO_2_^15,20^. The animal was sacrificed, and the brain was removed and immersed in ice-cold ACSF. Sagittal slices (400 μm) were prepared with a VT1000S vibratome (Leica Microsystems) and incubated at 35 °C for 20 minutes in oxygenated ACSF. After 2 h recovery at room temperature, slices were transferred to a recording chamber and perfused with ACSF (35 °C, 1.7 mL/min). Electrical stimulation (100 μs duration) of Shaffer collateral was achieved by a bipolar tungsten stimulating electrode (WPI, USA) placed in the stratum radiatum. Field excitatory post-synaptic potentials (fEPSPs) in CA1 stratum radiatum were recorded by a micropipette (1–3 MΩ) filled with ACSF. Baseline responses were obtained every 30 s with a stimulation intensity that yielded a half-maximal slope (mV/ms) response. After achievement of a 10 min stable baseline, a theta-burst stimulation (TBS) was delivered. The protocol of TBS stimulation consisted of 5 bursts at 5 Hz, each burst consisted of 4 pulses at 100 Hz^15,20^. Data, filtered at 0.1 Hz and 600 Hz and sampled at 25 kHz were acquired with a patch-clamp amplifier (Multiclamp 700B, Molecular Devices), and analyzed using pClamp 10.2 software (Molecular Devices).

For DG LTP mice were anesthetized with isoflurane and transcardially perfused with an ice-cold ACSF solution containing (in mM): 119 NaCl, 2.5 KCl, 1.3 MgSO_4_, 1 NaH_2_PO_4_, 26 NaHCO_3_, 2.5 CaCl_2_, and 10 glucose (pH 7.4), oxygenated with 95% O_2_ and 5% CO_2_^6^. The animal was sacrificed, and the brain was removed and immersed in ice-cold ACSF. Transverse hippocampal slices (400 μm) were prepared using a Microm HM 650 V vibratome equipped with Microm CU 65 cooling unit (Thermo Scientific) and incubated at 32 °C for 30 minutes in oxygenated ACSF. Slice were then transferred into a holding chamber containing oxygenated ACSF and incubated for at least 90 minutes at room temperature before recording. The slices were transferred into a recording chamber and continuously perfused with oxygenated ACSF (2 ml/min) at 31 °C ± 1 °C. Afferent fibers on the media perforant path (MPP) were stimulated using a bipolar tungsten stimulating electrode (WPI, USA), and fEPSPs were recorded using glass capillary electrodes (4–5 MΩ) filled with ACSF positioned in the medial molecular layer of the upper blade of the DG. Baseline recordings were conducted with a frequency of 1 test stimulus every 30 seconds. Only slices showing fEPSPs of 1 mV in amplitude or higher were selected for experiments. LTP was induced through a tetanic stimulation that consisted of four 100-Hz trains of 0.5 seconds each repeated every 30 seconds. After induction, the responses were recorded every 30 seconds for at least 60 minutes. The data were 0.1–1000 Hz band-pass filtered and amplified using a DAM 80 amplifier (WPI), digitized at a sampling rate of 25 kHz using a Digidata 1440 A (Molecular Devices) running with Clampex software (Molecular Devices). The Clampfit software (Molecular Devices) was used for data analysis.

#### Patch-clamp recordings

Whole-cell patch-clamp recordings from CA1 pyramidal neurons were performed using Multiclamp 700B/Digidata1440A system (Molecular Devices, Sunnyvale CA, USA) and an upright Olympus BX51WI microscope (Olympus) equipped with Nomarski optics. After anesthetization with isoflurane, transverse hippocampal slices (400 μm) were cut using a Microm HM 650 V microtome equipped with Microm CU 65 cooling unit (Thermo Fisher Scientific). Slices were cut at 2 °C in a solution containing (in mM): 87 NaCl, 25 NaHCO_3_, 2.5 KCl, 0.5 CaCl_2_, 7 MgCl_2_, 25 glucose, and 75 sucrose saturated with 95% O_2_ and 5% CO_2_. After cutting the slices were left to recover for 1 hour at 35 °C and for another 2 hours at room temperature in recording solution. Experiments were performed at 22–24 °C. The extracellular solution used for recordings contained (in mM): 125 NaCl, 25 NaHCO_3_, 25 glucose, 2.5 KCl, 1.25 NaH_2_PO_4_, 2 CaCl_2_, and 1 MgCl_2_ (bubbled with 95% O_2_-5% CO_2_). All experiments were performed at a holding potential Vh = −70 mV in the presence of 0.3 μM TTX (Tocris Bioscience). Miniature excitatory postsynaptic currents (mEPSP) were recorded with an internal solution containing (in mM): 126 K gluconate, 4 NaCl, 1 MgSO_4_, 0.02 CaCl_2_, 0.1 BAPTA, 15 Glucose, 5 HEPES, 3 ATP, 0.1 GTP (pH 7.3) in the presence of 30 μM bicuculline and 5 μM CGP55845 (Tocris Bioscience) to block GABA_A_ and GABA_B_ receptors, respectively. Miniature inhibitory postsynaptic currents (mIPSP) were recorded with a high-chloride intracellular solution containing (in mM): 126 KCl, 4 NaCl, 1 MgSO_4_, 0.02 CaCl_2_, 0.1 BAPTA, 15 Glucose, 5 HEPES, 3 ATP and 0.1 GTP (pH 7.3) in the presence of 50 μM D-AP5 and 10 μM CNQX (Tocris Bioscience) to block NMDA and non-NMDA receptors, respectively. Somatic access resistance was monitored continuously, and cells with access resistance larger than 15 MΩ and/or a holding current larger than −200 pA were excluded. All patch-clamp data were acquired with Clampex 10.2 and analyzed offline with Clampfit 10.2 (pClamp, Molecular Devices) and MiniAnalysis (Synaptosoft).

### Statistical analysis

The results are presented as the means ± SEM. The statistical analysis was performed using SigmaPlot (Systat) and GraphPad (Prism) software. Where appropriate, the statistical significance was assessed using the following parametric test: two-tailed unpaired t-test, two-way ANOVA or two-way repeated measure ANOVA followed by all pairwise Turkey’s *post hoc* test. In case normal distribution or equal variance assumptions were not valid, statistical significance was evaluated using Mann-Whitney test (non-parametric) or by two-way ANOVA on ranked transformed data. *P* values < 0.05 were considered significant.

### Ethical approval declaration

A veterinarian was employed to monitor health and comfort of the animals. Mice were housed in a temperature-controlled room with a 12:12 hour dark/light cycle with *ad libitum* access to water and food. All animal experiments were performed in accordance with the guidelines established by the European Community Council Directive 2010/63/EU of September 22^nd^, 2010 and were approved by the Italian Ministry of Health (authorization n°: 216/2012-B and 658/2016-PR).

## Electronic supplementary material

Supplementary information
