# Supplementary material for: Aerobic exercise and a BDNF-mimetic therapy rescue learning and memory in a mouse model of Down syndrome
Source: Sci Rep. 2017 Dec 4;7:16825. doi: 10.1038/s41598-017-17201-8 (PMC5715062; doi:10.1038/s41598-017-17201-8)
Supplement: Supplementary file 1 — Supplementary information [file 41598_2017_17201_MOESM1_ESM.pdf]

Supplementary information for:

Aerobic exercise and a BDNF-mimetic therapy rescue learning and memory in a mouse model of Down syndrome

Martina Parrini[1], Diego Ghezzi[1†], Gabriele Deidda[1§], Lucian Medrihan[1^], Enrico Castroflorio[2], Micol Alberti[1], Pietro Baldelli[2,3], Laura Cancedda[1] and Andrea Contestabile[1*].

[1] Department of Neuroscience and Brain Technologies, Istituto Italiano di Tecnologia, Genova, Italy.

[2] Center for Synaptic Neuroscience, Istituto Italiano di Tecnologia, Genova, Italy.

[3] Department of Experimental Medicine, University of Genova, Italy.

[†] Present address: Medtronic Chair in Neuroengineering, Center for Neuroprosthetics, Institute of Bioengineering, School of Engineering, École Polytechnique Fédérale de Lausanne, Switzerland

^ Present address: Laboratory of Molecular and Cellular Neuroscience, Rockefeller University, NY, USA.

[§] Present address: School of Biosciences, Cardiff University, UK.

*Corresponding author, andrea.contestabile@iit.it

Supplementary text and figures:

A

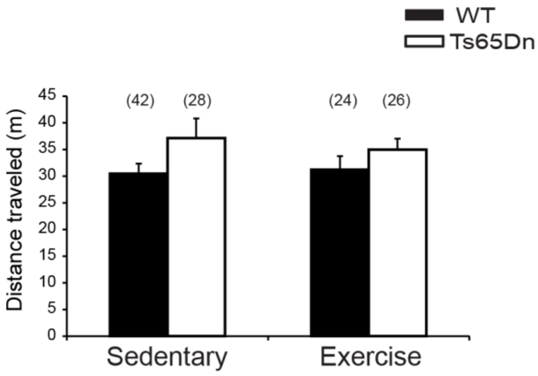

Supplementary Figure 1

Supplementary Figure 1. Physical exercise had no effect on general motor activity. **(A)** Distance traveled in the empty arena was not different across genotype and treatments. Two-way ANOVA on ranked transformed data: genotype [$F_{1,116}$ = 4.460, P = 0.037], treatment [$F_{1,116}$ = 0.038, P = 0.845], genotype x treatment [$F_{1,116}$ = 0.015, P = 0.901]. Tukey *post hoc* test following two-way ANOVA did not reach statistical significance for any experimental group. Number in parenthesis indicates the number of mice tested for each experimental group.

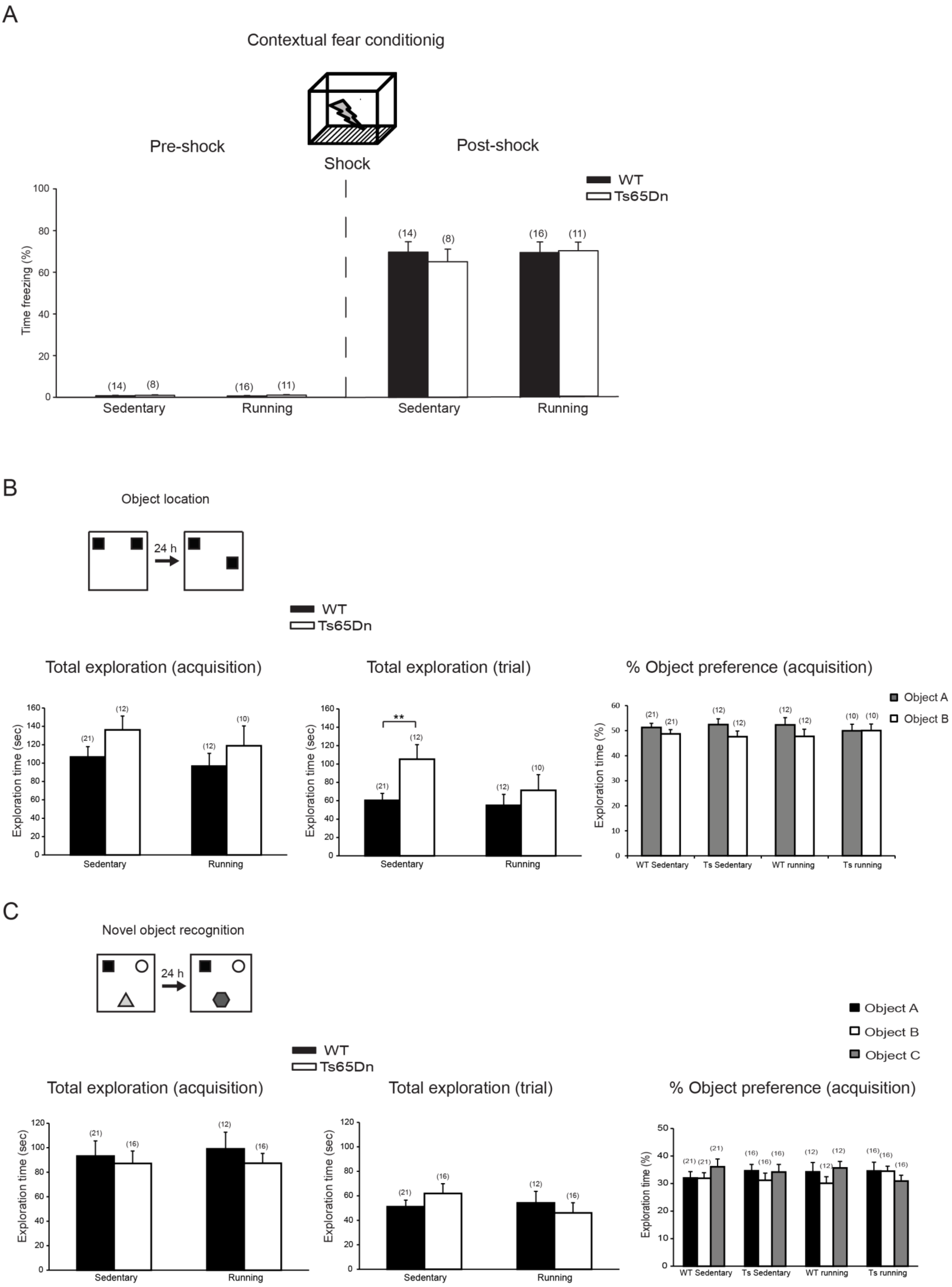

Supplementary Figure 2

Supplementary Figure 2. **(A)** Physical exercise had no effect on the freezing time before (left) or immediately after (right) the electric foot shock during the conditioning session in both Ts65Dn and WT mice. Pre-shock two-way ANOVA on ranked transformed data: genotype [$F_{1,45}$=1.964, P=0.168]; treatment [$F_{1,45}$=0.0661, P=0.798]; genotype x treatment [$F_{1,45}$=0.00002, P=0.996]. Post-shock two-way ANOVA: genotype [$F_{1,45}$=0.171, P=0.681]; treatment [$F_{1,45}$= 0.169, P=0.683]; genotype x treatment [$F_{1,45}$=0.194, P=0.661]. **(B)** *Left:* in the OL test the total exploration time during the acquisition phase was not significantly different across genotype and treatment. Two-way ANOVA: genotype [$F_{1,51}$=5.017, P=0.029]; treatment [$F_{1,51}$=0.641, P=0.427]; genotype x treatment [$F_{1,51}$=0.0223, P=0.882]. *Center*: the total exploration time during the trial phase was slightly increased in sedentary Ts65Dn mice compared to WT, however no significant difference was observed in Ts65Dn running mice compared to WT. Two-way ANOVA: genotype [$F_{1,51}$=9.164, P=0.004]; treatment [$F_{1,51}$=2.453, P=0.123]; genotype x treatment [$F_{1,51}$=0.914, P=0.349]. **P<0.01, Tukey post hoc test. *Right*: the percentage of time spent exploring the two objects during the acquisition phase was not statistically different across the experimental groups. Two-way ANOVA: genotype [$F_{1,102}$<0.0001, P=1.000]; treatment [$F_{1,102}$=1.331, P=0.268]; genotype x treatment [$F_{1,102}$=0.4014, P=0.753]. **(C)** *Left:* in the NOR test the total exploration time during the acquisition phases was not significantly different across genotype and treatment. Two-way ANOVA on ranked transformed data: genotype [$F_{1,61}$=0.585; P=0.447]; treatment [$F_{1,61}$=0.548; P=0.462]; genotype x treatment [$F_{1,61}$=0.0997; P=0.753]. *Center*: the total exploration time during the trial phase was not significantly different across genotype and treatment. Two-way ANOVA on ranked transformed data: genotype [$F_{1,61}$=0.0226; P=0.881]; treatment [$F_{1,61}$=1.892; P=0.174]; genotype x treatment [$F_{1,61}$=2.047; P=0.158]. *Right*: the percentage of time spent exploring the three objects during the acquisition phase was not statistically different across the experimental groups. Two-way ANOVA: genotype [$F_{1,187}$=0.231; P=0.645]; treatment [$F_{1,187}$=0.718; P=0.629]; genotype x treatment [$F_{1,187}$=0.895; P=0.521]. Number in parenthesis indicates the number of mice tested for each experimental group.

Asymmetric synapses (Glutamatergic)

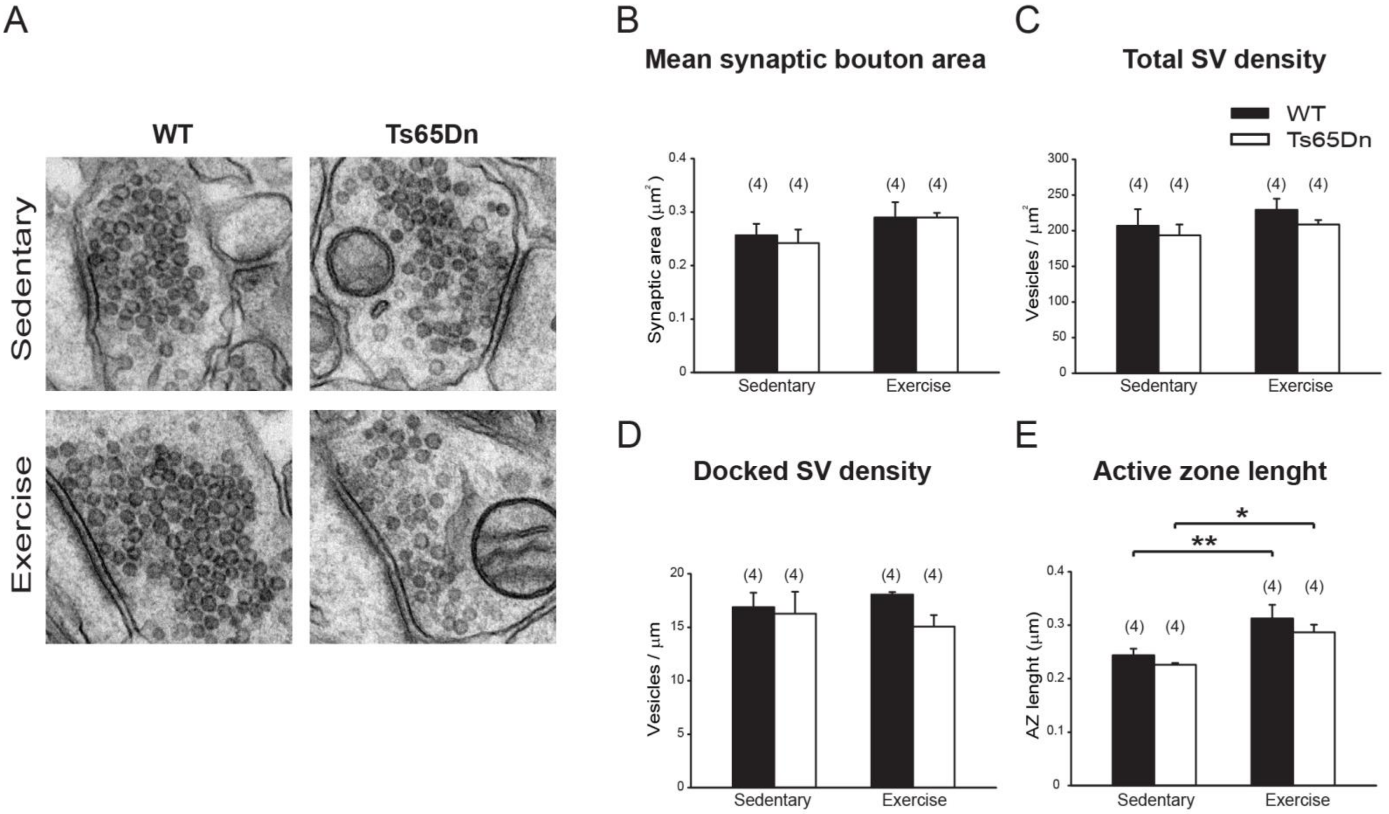

Symmetric synapses (GABAergic)

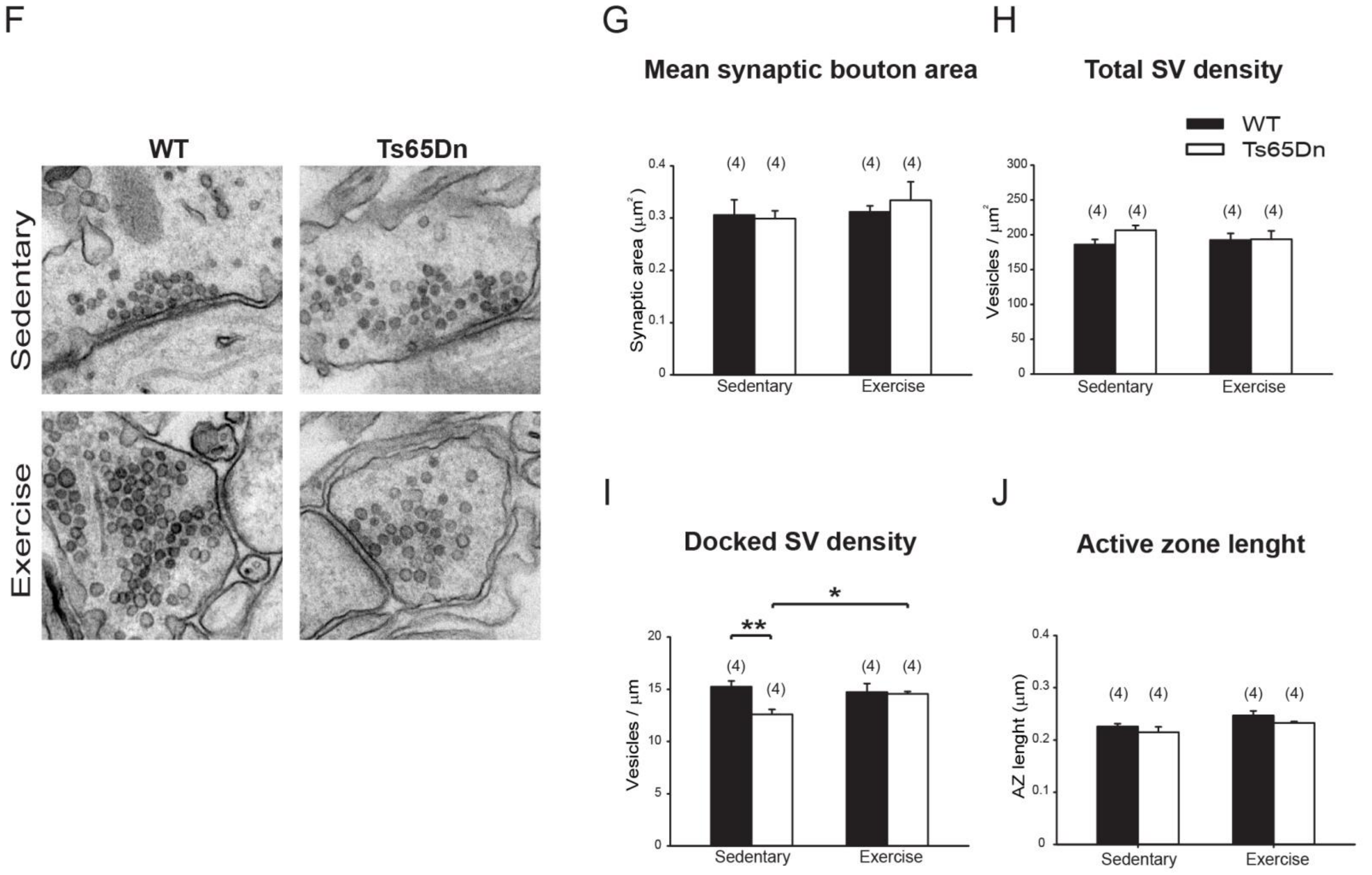

Supplementary Figure 3

Supplementary Figure 3. Synaptic ultrastructural morphometric analysis in the hippocampal CA1 region. **(A)** Representative TEM images showing asymmetric (glutamatergic) synapses. **(B)** Mean asymmetric synaptic bouton area was not significantly different across genotype and treatment. Two-way ANOVA: genotype [$F_{1,15}$=0.108, P=0.748]; treatment [$F_{1,15}$=3.218, P=0.098]; genotype x treatment [$F_{1,15}$=0.097, P=0.761]. **(C)** Total synaptic vesicles (SV) density in asymmetric synapses was not significantly different across genotype and treatment. Two-way ANOVA: genotype [$F_{1,15}$=1.078, P=0.320]; treatment [$F_{1,15}$=1.333, P=0.271]; genotype x treatment [$F_{1,15}$=0.045, P=0.836]. **(D)** The density of docked SV in asymmetric synapses was not significantly different across genotype and treatment. Two-way ANOVA: genotype [$F_{1,15}$=1.797, P=0.205]; treatment [$F_{1,15}$<0.0001, P=0.997]; genotype x treatment [$F_{1,15}$= 0.779, P=0.395]. **(E)** In asymmetric synapses the length of the active zone was increased by exercise in both WT and Ts65Dn mice. Two-way ANOVA: genotype [$F_{1,15}$=1.889, P=0.194]; treatment [$F_{1,15}$=16.940, P=0.001]; genotype x treatment [$F_{1,15}$=0.0745, P=0.790]. **(F)** Representative TEM images showing symmetric (GABAergic) synapses. **(G)** Mean symmetric synaptic bouton area was not significantly different across genotype and treatment. Two-way ANOVA: genotype [$F_{1,15}$=0.0952, P=0.763]; treatment [$F_{1,15}$=0.679, P=0.426]; genotype x treatment [$F_{1,15}$=0.353, P=0.564]. **(H)** Total SV density in symmetric synapses was not significantly different across genotype and treatment. Two-way ANOVA: genotype [$F_{1,15}$=1.379, P=0.263]; treatment [$F_{1,15}$=0.124, P=0.731]; genotype x treatment [$F_{1,15}$=1.145, P=0.306]. **(I)** The density of docked SV in symmetric synapses was reduced in sedentary Ts65Dn mice and significantly restored by exercise. Two-way ANOVA: genotype [$F_{1,15}$=6.265, P=0.028]; treatment [$F_{1,15}$=1.633, P=0.225]; genotype x treatment [$F_{1,15}$=4.922, P=0.047]. **(J)** In symmetric synapses the length of the active zone was not significantly different across genotype and treatment. Two-way ANOVA: genotype [$F_{1,15}$=2.777, P=0.121]; treatment [$F_{1,15}$=6.939, P=0.022]; genotype x treatment [$F_{1,15}$=0.0233, P=0.881]. *P<0.05, **P<0.01, Tukey *post hoc* test following two-way ANOVA. Number in parenthesis indicates the number of mice tested for each experimental group.

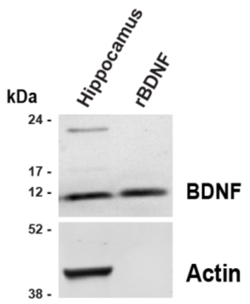

Supplementary Figure 4

Supplementary Figure 4. Specificity of the anti-BDNF antibody used for western blot analysis. Representative immunoblot with anti-BDNF antibody on hippocampal protein extracts or recombinant BDNF (rBDNF). Actin was used as loading control and is absent in rBDNF line.

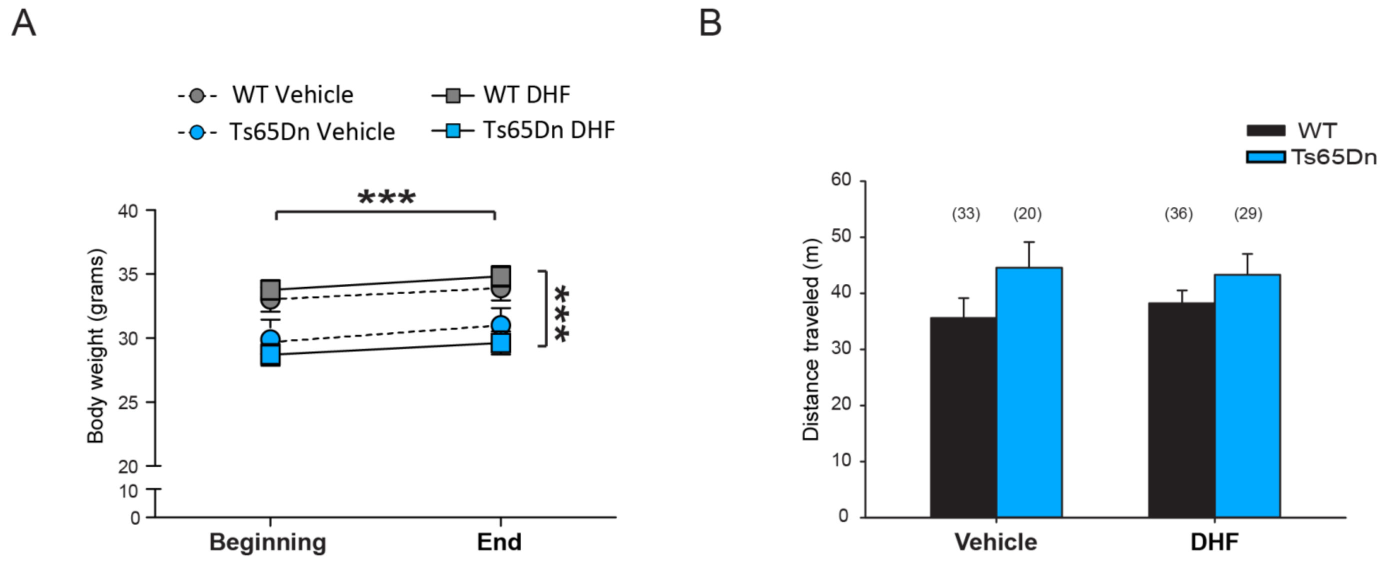

Supplementary Figure 5

Supplementary Figure 5. DHF treatment had no effect on body weight gain and general motor activity. **(A)** Mice body weight was measured at the beginning and at the end (4 weeks later) of chronic DHF or vehicle treatment. As previously reported (Costa, et al. 2010), mean body weight was greater in WT than in Ts65Dn mice (P<0.001). All groups of mice showed comparable and significant weight gain (P<0.001 for all groups) during the 4-week treatment period independently of genotype. Two-way repeated measure ANOVA on ranked-transformed data: genotype [$F_{3,114}$ = 8.509, P < 0.001], time-point [$F_{1,114}$ = 59.318, P < 0.001], genotype x time-point [$F_{3,114}$ = 0.407, P = 0.748]. ***P<0.001, Tukey *post hoc* test. **(B)** Motor activity was evaluated by measuring the distance traveled in the empty arena by mice after chronic DHF or vehicle treatment. Distance traveled was not different across genotype and treatments. Two-way ANOVA: genotype [$F_{1,114}$ = 4.058, P = 0.046], treatment [$F_{1,114}$ = 0.039, P = 0.843], genotype x treatment [$F_{1,114}$ = 0.313, P = 0.577]. Tukey *post hoc* test following two-way ANOVA did not reach statistical significance for any experimental group. Number in parenthesis indicates the number of mice tested for each experimental group.

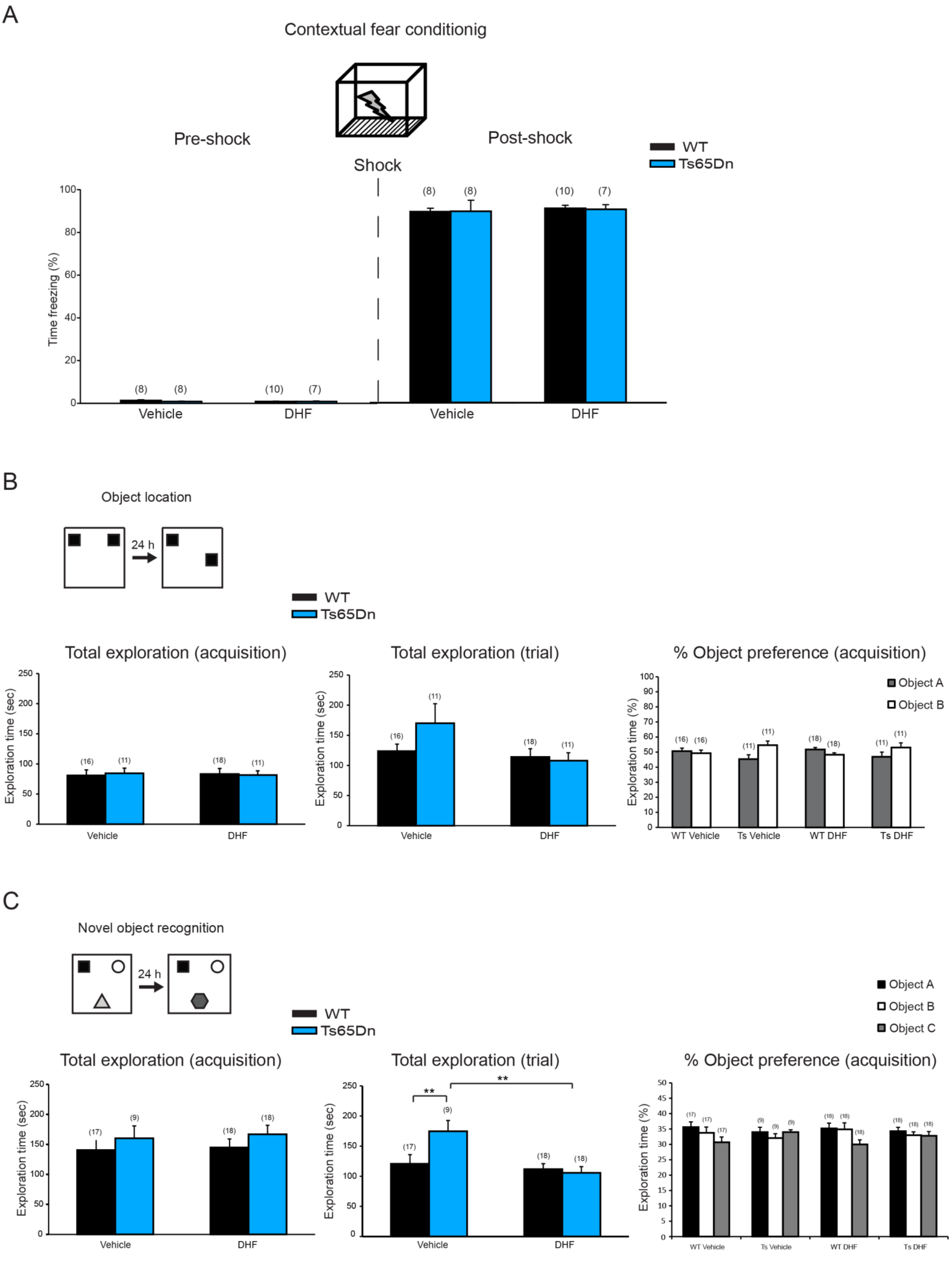

Supplementary Figure 6

Supplementary Figure 6. **(A)** DHF treatment had no effect on the freezing time before (left) or immediately after (right) the electric foot shock during the conditioning session in both Ts65Dn and WT mice. Pre-shock two-way ANOVA: genotype [$F_{1,29}$=1.357, P=0.254]; treatment [$F_{1,29}$=0.505, P=0.483]; genotype x treatment [$F_{1,29}$=1.665, P=0.207]. Post-shock two-way ANOVA: genotype [$F_{1,29}$=0.576, P=0.454]; treatment [$F_{1,29}$=0.140, P=0.711]; genotype x treatment [$F_{1,29}$=2.394, P=0.133]. **(B)** *Left:* in the OL the total exploration time during the acquisition phase was not significantly different across genotype and treatment. Two-way ANOVA: genotype [$F_{1,52}$=0.0084, P=0.927]; treatment [$F_{1,52}$=0.0005, P=0.981]; genotype x treatment [$F_{1,52}$=0.0803, P=0.778]. *Center*: the total exploration time during the trial phase was not significant different across genotype and treatment. Two-way ANOVA: genotype [$F_{1,52}$=0.215, P=0.645]; treatment [$F_{1,52}$=3.764, P=0.058]; genotype x treatment [$F_{1,52}$=0.972, P=0.329]. *Right*: the percentage of time spent exploring the two objects during the acquisition phase was not statistically different across the experimental groups. Two-way ANOVA: genotype [$F_{1,102}$<0.0001, P=1.000]; treatment [$F_{1,102}$=1.108, P=0.349]; genotype x treatment [$F_{1,102}$=2.680, P=0.051]. Number in parenthesis indicates the number of mice tested for each group experimental. **(C)** *Left:* in the NOR test the total exploration time during the acquisition was not significantly different across genotype and treatment. Two-way ANOVA: genotype [$F_{1,58}$=1.526; P=0.222]; treatment [$F_{1,58}$=0.101; P=0.752]; genotype x treatment [$F_{1,58}$=0.0059; P=0.939]. *Center*: the total exploration time during the trial phase was slightly increased in vehicle-treated Ts65Dn mice compared to WT, however no significant difference was observed in Ts65Dn mice treated with DHF compared to WT. Two-way ANOVA: genotype [$F_{1,58}$=3.301; P=0.074]; treatment [$F_{1,58}$=8.720; P=0.005]; genotype x treatment [$F_{1,58}$=5.193; P=0.026]. *Right*: the percentage of time spent exploring the three objects was not statistically different across groups. Two-way ANOVA on ranked transformed data: genotype [$F_{1,174}$=0.110; P=0.741]; treatment [$F_{1,174}$=1.396; P=0.228]; genotype x treatment [$F_{1,174}$=1.141; P=0.340].The number in parenthesis indicate the number of mice tested for each experimental group. **p<0.01, Tukey *post hoc* test following two-way ANOVA.

Full-length blots for Figure 4B

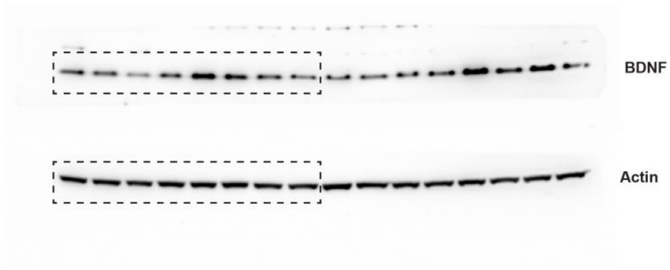

Full-length blots for Figure 5A

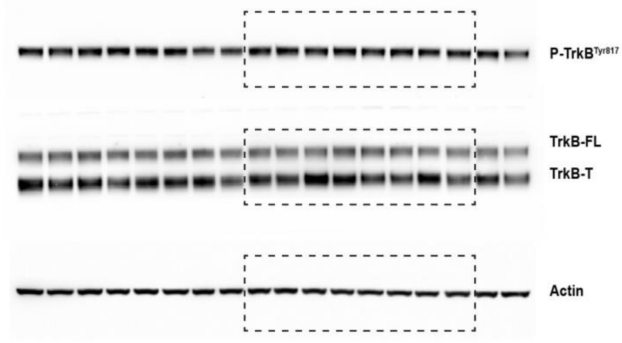

Full-length blots for Figure 5B

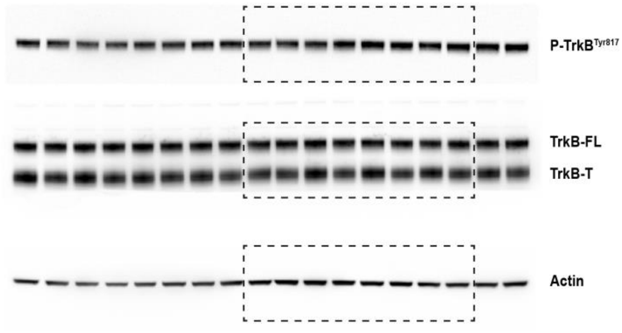

Full-length blots for Figure 5C

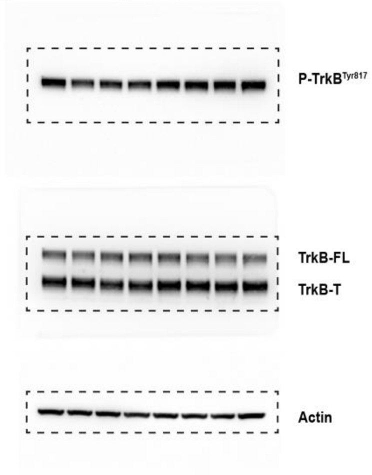

Full-length blots for Figure 5D

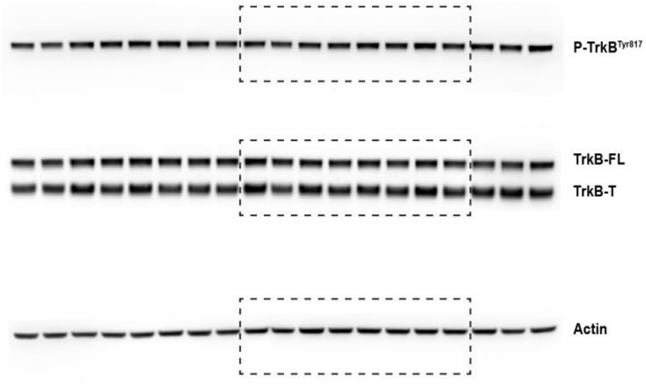

Full-length blots for Figure 7B

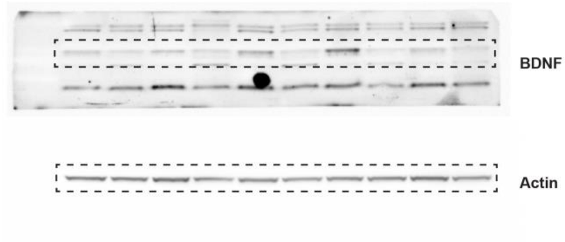

Full-length blots for Supplemenatry Figure 4

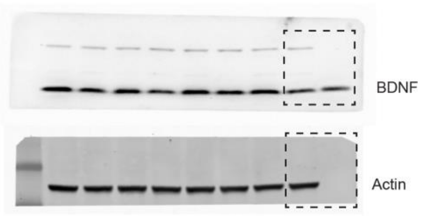

Supplementary Figure 7

Supplementary Fig. 7 Full-length blot images corresponding to the cropped western blot presented in Figure 4, 5, 7 and Supplementary Figure 4.

Supplementary Table 1. Primer sequences for RT-qPCR analysis.

Gene symbol	Gene name	Organism	Accession number	Forward primer (*5′-3′*)	Reverse primer (*5′-3′*)
ACTB	Actin beta	Homo Sapiens	NM_001101	CAGCAAGCAGGAGTATGAC	GAAAGGGTGTAACGCAACT
GAPDH	Glyceraldehyde-3-phosphate dehydrogenase	Homo Sapiens	NM_001256799	AATGAAGGGGTCATTGATGG	AAGGTGAAGGTCGGAGTCAA
PPIA	Peptidylprolyl isomerase A	Homo Sapiens	NM_021130	TTCTGCTGTCTTTGGGACCT	CACCGTGTTCTTCGACATTG
BDNF (CDS)	Brain-derived neurotrophic factor	Homo Sapiens	NM_001143805<br>NM_001143806<br>NM_001143807<br>NM_001143808<br>NM_001143809<br>NM_00114381<br>NM_001143811<br>NM_00114381<br>NM_001143814<br>NM_00114381<br>NM_001709<br>NM_170731<br>NM_170732<br>NM_170733<br>NM_170734<br>NM_170735	CGAGACCAAGTGCAATCC	TTATGAATCGCCAGCCAAT
Actb	Actin beta	Mus Musculus	NM_007393	AAGTGGTTACAGGAAGTCC	ATAATTTACACAGAAGCAATGC
Gapdh	Glyceraldehyde-3-phosphate dehydrogenase	Mus Musculus	NM_008084	GAACATCATCCCTGCATCCA	CCAGTGAGCTTCCCGTTCA
Ppia	Peptidylprolyl isomerase A	Mus Musculus	NM_008907	CACTGTCGCTTTTCGCCGCTTG	TTTCTGCTGTCTTTGGAACTTTGTCTGC
Bdnf (Exon I)	Brain-derived neurotrophic factor	Mus Musculus	NM_007540	TGGTAACCTCGCTCATTCATTAGA	CCCTTCGCAATATCCGCAAAG
Bdnf (Exon II)	Brain-derived neurotrophic factor	Mus Musculus	NM_001048139	CTTTTCCTCGCTGTCAAG	TTGCCAAGAGTCTATTCC
Bdnf (Exon III)	Brain-derived neurotrophic factor	Mus Musculus	NM_001285419	TCACGATCCTCGATGGATAGTTCT	AGGGAGTGGAGCGCAGTC
Bdnf (Exon IV)	Brain-derived neurotrophic factor	Mus Musculus	NM_001048141	CAAATGGAGCTTCTCGCTGAAGGC	GTGGAAATTGCATGGCGGAGGTAA
Bdnf (Exon V)	Brain-derived neurotrophic factor	Mus Musculus	NM_001285420	ACCATAACCCCGCACACTCTGT	GCACCTTCCCGCACCACAAA
Bdnf (Exon VI)	Brain-derived neurotrophic factor	Mus Musculus	NM_001048142	CTTCAACTGCCACCACTG	CATTGTTGTCACGCTTCTG
Bdnf (Exon VII)	Brain-derived neurotrophic factor	Mus Musculus	NM_001285417	GCTTACTTACAGGTCCAAGGTCAA	GTCCTGGAGTTCCGCAGAC
Bdnf (CDS)	Brain-derived neurotrophic factor	Mus Musculus	NM_007540<br>NM_001048139<br>NM_001048141<br>NM_001048142<br>NM_001285416<br>NM_001285417<br>NM_001285418<br>NM_001285419<br>NM_001285420<br>NM_001285421<br>NM_001285422	ATTACCTGGATGCCGCAAA	TAATACTGTCACACACGCTCA

Supplementary Table 2. Calibration curves parameters, PCR reaction efficiency and amplicon information for RT-qPCR analysis.

**Gene symbol**	**Organism**	**Amplicon length (bp)**	**Final primer concentration (μM)**	**Calibration curve (slope / R^2)**	**Calculated PCR efficiency (%)**	**Primer dimers**
ACTB	Homo Sapiens	91	0.2	-3.242 / 0.997	103.4	Not detected
GAPDH	Homo Sapiens	108	0.2	-3.493 / 0.999	93.3	Only in NTC samples
PPIA	Homo Sapiens	91	0.2	-3.446 / 0.997	95.1	Not detected
BDNF (CDS)	Homo Sapiens	149	0.2	-3.282 / 0.999	101.7	Not detected
Actb	Mus Musculus	123	0.1	-3.130 / 0.986	108.7	Not detected
Gapdh	Mus Musculus	77	0.2	-3.370 / 0.999	98.0	Only in NTC samples
Ppia	Mus Musculus	133	0.2	-3.321 / 0.994	100.0	Only in NTC samples
Bdnf (Exon I)	Mus Musculus	172	0.2	-3.450 / 0.999	94.9	Not detected
Bdnf (Exon II)	Mus Musculus	200	0.1	-3.175 / 0.998	106.5	Not detected
Bdnf (Exon III)	Mus Musculus	150	0.2	-3.716 / 0.994	85.3	Not detected
Bdnf (Exon IV)	Mus Musculus	57	0.2	-3.584 / 0.999	90.1	Not detected
Bdnf (Exon V)	Mus Musculus	73	0.2	-3.536 / 0.997	91.8	Not detected
Bdnf (Exon VI)	Mus Musculus	87	0.2	-3.573 / 0.999	90.5	Only in NTC samples
Bdnf (Exon VII)	Mus Musculus	93	0.2	-3.503 / 0.996	92.9	Not detected
Bdnf (CDS)	Mus Musculus	90	0.2	-3.315 / 0.999	100.3	Not detected

Supplementary Table 3. Human sample information.

Case number	Disorder	Age (years)	Gender
1276	DS	13	M
5277	DS	19	M
M1960M	DS	19	M
5341	DS	25	M
5005	DS	39	F
4925	Control	13	M
4782	Control	18	M
1841	Control	19	M
605	Control	25	M
5606	Control	35	F

Supplementary Table 4. List of mice used in the study.

			Behavior			IHC					
Mouse #	Genotype	Treatment	NOR	OL	CFC	BrdU 24hr	BrdU 4w	VGAT/VGLUT1	EPhy	EF	Bioch
1	WT	sedentary	*			*		*			
2	WT	sedentary	*			*					
3	WT	sedentary	*			*					
4	WT	sedentary	*			*					
5	WT	sedentary	*			*					
6	WT	sedentary	*			*		*			
7	WT	sedentary	*				*				
8	WT	sedentary	*				*				
9	WT	sedentary	*				*				
10	WT	sedentary	*				*	*	*		
11	WT	sedentary	*				*	*	*		
12	WT	sedentary	*				*				
13	WT	sedentary	*				*	*	*		
14	WT	sedentary	*				*	*	*		
15	WT	sedentary	*				*				
16	WT	sedentary	*				*				
17	WT	sedentary	*								
18	WT	sedentary	*								
19	WT	sedentary	*								
20	WT	sedentary	*								
21	WT	sedentary	*								

22	WT	sedentary	*								
23	WT	sedentary	*								
24	WT	sedentary	*								
25	WT	sedentary	*								
26	WT	sedentary	*								
27	WT	sedentary	*								
28	WT	sedentary	*								
29	WT	sedentary			*						
30	WT	sedentary			*						
31	WT	sedentary			*						
32	WT	sedentary			*						
33	WT	sedentary			*						
34	WT	sedentary			*						
35	WT	sedentary			*						
36	WT	sedentary			*						
37	WT	sedentary			*						
38	WT	sedentary			*						
39	WT	sedentary			*						
40	WT	sedentary			*						
41	WT	sedentary			*						
42	WT	sedentary			*						
43	WT	sedentary		*							
44	WT	sedentary		*							
45	WT	sedentary		*							
46	WT	sedentary		*							
47	WT	sedentary		*							
48	WT	sedentary		*							
49	WT	sedentary		*							
50	WT	sedentary		*							
51	WT	sedentary		*							
52	WT	sedentary		*							
53	WT	sedentary		*							
54	WT	sedentary		*							
55	WT	sedentary		*							
56	WT	sedentary		*							
57	WT	sedentary		*							
58	WT	sedentary		*							
59	WT	sedentary		*							
60	WT	sedentary		*							
61	WT	sedentary		*							
62	WT	sedentary		*							
63	WT	sedentary		*							
64	WT	sedentary								*	
65	WT	sedentary								*	
66	WT	sedentary								*	
67	WT	sedentary								*	
68	WT	sedentary								*	

69	WT	sedentary								*	
70	WT	sedentary								*	
71	WT	sedentary								*	
72	WT	sedentary								*	
73	WT	sedentary								*	
74	WT	sedentary								*	
75	WT	sedentary								*	
76	WT	sedentary								*	
77	WT	sedentary								*	
78	WT	sedentary								*	
79	WT	sedentary								*	
80	WT	sedentary									*
81	WT	sedentary									*
82	WT	sedentary									*
83	WT	sedentary									*
84	WT	sedentary									*
85	WT	sedentary									*
86	WT	sedentary									*
87	WT	sedentary									*
88	WT	sedentary									*
89	WT	sedentary									*
90	WT	sedentary									*
91	WT	sedentary									*
92	Ts65Dn	sedentary	*			*					
93	Ts65Dn	sedentary	*			*		*	*		
94	Ts65Dn	sedentary	*			*					
95	Ts65Dn	sedentary	*			*		*			
96	Ts65Dn	sedentary	*			*		*	*		
97	Ts65Dn	sedentary	*			*					
98	Ts65Dn	sedentary	*			*		*			
99	Ts65Dn	sedentary	*				*	*	*		
100	Ts65Dn	sedentary	*				*	*	*		
101	Ts65Dn	sedentary	*				*	*			
102	Ts65Dn	sedentary	*				*	*			
103	Ts65Dn	sedentary	*				*	*			
104	Ts65Dn	sedentary	*								
105	Ts65Dn	sedentary	*								
106	Ts65Dn	sedentary	*								
107	Ts65Dn	sedentary	*								
108	Ts65Dn	sedentary	*								
109	Ts65Dn	sedentary			*						
110	Ts65Dn	sedentary			*						
111	Ts65Dn	sedentary			*						
112	Ts65Dn	sedentary			*						
113	Ts65Dn	sedentary			*						
114	Ts65Dn	sedentary			*						
115	Ts65Dn	sedentary			*						

116	Ts65Dn	sedentary		*	*						
117	Ts65Dn	sedentary		*							
118	Ts65Dn	sedentary		*							
119	Ts65Dn	sedentary		*							
120	Ts65Dn	sedentary		*							
121	Ts65Dn	sedentary		*							
122	Ts65Dn	sedentary		*							
123	Ts65Dn	sedentary		*							
124	Ts65Dn	sedentary		*							
125	Ts65Dn	sedentary		*							
126	Ts65Dn	sedentary		*							
127	Ts65Dn	sedentary		*							
128	Ts65Dn	sedentary								*	
129	Ts65Dn	sedentary								*	
130	Ts65Dn	sedentary								*	
131	Ts65Dn	sedentary								*	
132	Ts65Dn	sedentary								*	
133	Ts65Dn	sedentary								*	
134	Ts65Dn	sedentary								*	
135	Ts65Dn	sedentary								*	
136	Ts65Dn	sedentary								*	
137	Ts65Dn	sedentary								*	
138	Ts65Dn	sedentary								*	
139	Ts65Dn	sedentary								*	
140	Ts65Dn	sedentary									*
141	Ts65Dn	sedentary									*
142	Ts65Dn	sedentary									*
143	Ts65Dn	sedentary									*
144	Ts65Dn	sedentary									*
145	Ts65Dn	sedentary									*
146	Ts65Dn	sedentary									*
147	Ts65Dn	sedentary									*
148	Ts65Dn	sedentary									*
149	Ts65Dn	sedentary									*
150	Ts65Dn	sedentary									*
151	Ts65Dn	sedentary									*
152	WT	exercise	*			*					
153	WT	exercise	*			*					
154	WT	exercise	*			*					
155	WT	exercise	*			*		*			
156	WT	exercise	*			*					
157	WT	exercise	*			*					
158	WT	exercise	*			*		*	*		
159	WT	exercise	*			*		*			
160	WT	exercise	*				*	*			
161	WT	exercise	*				*	*			
162	WT	exercise	*				*				

163	WT	exercise	*				*				
164	WT	exercise		*			*	*	*		
165	WT	exercise		*			*	*	*		
166	WT	exercise		*			*				
167	WT	exercise		*			*				
168	WT	exercise		*			*	*	*		
169	WT	exercise	*								
170	WT	exercise	*								
171	WT	exercise			*						
172	WT	exercise			*						
173	WT	exercise			*						
174	WT	exercise			*						
175	WT	exercise			*						
176	WT	exercise			*						
177	WT	exercise			*						
178	WT	exercise			*						
179	WT	exercise			*						
180	WT	exercise			*						
181	WT	exercise			*						
182	WT	exercise			*						
183	WT	exercise			*						
184	WT	exercise			*						
185	WT	exercise			*						
186	WT	exercise			*						
187	WT	exercise		*							
188	WT	exercise		*							
189	WT	exercise		*							
190	WT	exercise		*							
191	WT	exercise		*							
192	WT	exercise		*							
193	WT	exercise		*							
194	WT	exercise		*							
195	WT	exercise		*							
196	WT	exercise		*							
197	WT	exercise		*							
198	WT	exercise		*							
199	WT	exercise								*	
200	WT	exercise								*	
201	WT	exercise								*	
202	WT	exercise								*	
203	WT	exercise								*	
204	WT	exercise								*	
205	WT	exercise								*	
206	WT	exercise								*	
207	WT	exercise								*	
208	WT	exercise								*	
209	WT	exercise								*	

210	WT	exercise								*	
211	WT	exercise									*
212	WT	exercise									*
213	WT	exercise									*
214	WT	exercise									*
215	WT	exercise									*
216	WT	exercise									*
217	WT	exercise									*
218	WT	exercise									*
219	WT	exercise									*
220	WT	exercise									*
221	WT	exercise									*
222	WT	exercise									*
223	Ts65Dn	exercise	*			*					
224	Ts65Dn	exercise	*			*					
225	Ts65Dn	exercise	*			*		*			
226	Ts65Dn	exercise	*			*					
227	Ts65Dn	exercise	*			*					
228	Ts65Dn	exercise	*			*		*			
229	Ts65Dn	exercise	*				*	*	*		
230	Ts65Dn	exercise	*				*				
231	Ts65Dn	exercise		*			*				
232	Ts65Dn	exercise	*				*	*	*		
233	Ts65Dn	exercise	*				*	*	*		
234	Ts65Dn	exercise		*			*	*	*		
235	Ts65Dn	exercise		*			*				
236	Ts65Dn	exercise	*								
237	Ts65Dn	exercise	*								
238	Ts65Dn	exercise	*								
239	Ts65Dn	exercise	*								
240	Ts65Dn	exercise	*								
241	Ts65Dn	exercise	*								
242	Ts65Dn	exercise	*								
243	Ts65Dn	exercise			*						
244	Ts65Dn	exercise			*						
245	Ts65Dn	exercise			*						
246	Ts65Dn	exercise			*						
247	Ts65Dn	exercise			*						
248	Ts65Dn	exercise			*						
249	Ts65Dn	exercise			*						
250	Ts65Dn	exercise			*						
251	Ts65Dn	exercise			*						
252	Ts65Dn	exercise		*	*						
253	Ts65Dn	exercise		*	*						
254	Ts65Dn	exercise		*							
255	Ts65Dn	exercise		*							
256	Ts65Dn	exercise		*							

257	Ts65Dn	exercise		*							
258	Ts65Dn	exercise		*							
259	Ts65Dn	exercise		*							
260	Ts65Dn	exercise		*							
261	Ts65Dn	exercise		*							
262	Ts65Dn	exercise								*	
263	Ts65Dn	exercise								*	
264	Ts65Dn	exercise								*	
265	Ts65Dn	exercise								*	
266	Ts65Dn	exercise								*	
267	Ts65Dn	exercise								*	
268	Ts65Dn	exercise								*	
269	Ts65Dn	exercise								*	
270	Ts65Dn	exercise								*	
271	Ts65Dn	exercise								*	
272	Ts65Dn	exercise								*	
273	Ts65Dn	exercise								*	
274	Ts65Dn	exercise									*
275	Ts65Dn	exercise									*
276	Ts65Dn	exercise									*
277	Ts65Dn	exercise									*
278	Ts65Dn	exercise									*
279	Ts65Dn	exercise									*
280	Ts65Dn	exercise									*
281	Ts65Dn	exercise									*
282	Ts65Dn	exercise									*
283	Ts65Dn	exercise									*
284	Ts65Dn	exercise									*
285	Ts65Dn	exercise									*
286	WT	vehicle	*								
287	WT	vehicle	*								
288	WT	vehicle	*								
289	WT	vehicle	*								
290	WT	vehicle	*								
291	WT	vehicle	*								
292	WT	vehicle	*		*						
293	WT	vehicle	*		*						
294	WT	vehicle	*		*						
295	WT	vehicle	*		*						
296	WT	vehicle	*		*						
297	WT	vehicle	*		*						
298	WT	vehicle	*		*						
299	WT	vehicle	*		*						
300	WT	vehicle	*								
301	WT	vehicle	*								
302	WT	vehicle	*								
303	WT	vehicle		*							
304	WT	vehicle		*							

305	WT	vehicle		*							
306	WT	vehicle		*							
307	WT	vehicle		*							
308	WT	vehicle		*							
309	WT	vehicle		*							
310	WT	vehicle		*							
311	WT	vehicle		*							
312	WT	vehicle		*							
313	WT	vehicle		*							
314	WT	vehicle		*							
315	WT	vehicle		*							
316	WT	vehicle		*							
317	WT	vehicle		*							
318	WT	vehicle		*							
319	WT	vehicle								*	
320	WT	vehicle								*	
321	WT	vehicle								*	
322	WT	vehicle								*	
323	WT	vehicle								*	
324	WT	vehicle								*	
325	WT	vehicle									*
326	WT	vehicle									*
327	WT	vehicle									*
328	WT	vehicle									*
329	WT	vehicle									*
330	WT	vehicle									*
331	WT	vehicle									*
332	WT	vehicle									*
333	WT	vehicle									*
334	WT	vehicle									*
335	WT	vehicle									*
336	WT	vehicle									*
337	WT	vehicle (acute)									*
338	WT	vehicle (acute)									*
339	WT	vehicle (acute)									*
340	WT	vehicle (acute)									*
341	WT	vehicle (acute)									*
342	WT	vehicle (acute)									*
343	WT	vehicle (acute)									*
344	WT	vehicle (acute)									*
345	WT	vehicle (acute)									*
346	WT	vehicle (acute)									*
347	WT	vehicle (acute)									*
348	WT	vehicle (acute)									*
349	WT	vehicle (acute)									*
350	WT	vehicle (acute)									*
351	WT	vehicle (acute)									*
352	WT	vehicle (acute)									*
353	Ts65Dn	vehicle	*		*						

354	Ts65Dn	vehicle	*		*						
355	Ts65Dn	vehicle	*		*						
356	Ts65Dn	vehicle	*		*						
357	Ts65Dn	vehicle	*		*						
358	Ts65Dn	vehicle	*		*						
359	Ts65Dn	vehicle	*		*						
360	Ts65Dn	vehicle	*		*						
361	Ts65Dn	vehicle	*								
362	Ts65Dn	vehicle		*							
363	Ts65Dn	vehicle		*							
364	Ts65Dn	vehicle		*							
365	Ts65Dn	vehicle		*							
366	Ts65Dn	vehicle		*							
367	Ts65Dn	vehicle		*							
368	Ts65Dn	vehicle		*							
369	Ts65Dn	vehicle		*							
370	Ts65Dn	vehicle		*							
371	Ts65Dn	vehicle		*							
372	Ts65Dn	vehicle		*							
373	Ts65Dn	vehicle								*	
374	Ts65Dn	vehicle								*	
375	Ts65Dn	vehicle									*
376	Ts65Dn	vehicle									*
377	Ts65Dn	vehicle									*
378	Ts65Dn	vehicle									*
379	Ts65Dn	vehicle									*
380	Ts65Dn	vehicle									*
381	Ts65Dn	vehicle									*
382	Ts65Dn	vehicle									*
383	Ts65Dn	vehicle									*
384	Ts65Dn	vehicle									*
385	Ts65Dn	vehicle									*
386	Ts65Dn	vehicle									*
387	Ts65Dn	vehicle (acute)									*
388	Ts65Dn	vehicle (acute)									*
389	Ts65Dn	vehicle (acute)									*
390	Ts65Dn	vehicle (acute)									*
391	Ts65Dn	vehicle (acute)									*
392	Ts65Dn	vehicle (acute)									*
393	Ts65Dn	vehicle (acute)									*
394	Ts65Dn	vehicle (acute)									*
395	Ts65Dn	vehicle (acute)									*
396	Ts65Dn	vehicle (acute)									*
397	Ts65Dn	vehicle (acute)									*
398	Ts65Dn	vehicle (acute)									*
399	Ts65Dn	vehicle (acute)									*
400	Ts65Dn	vehicle (acute)									*
401	Ts65Dn	vehicle (acute)									*
402	Ts65Dn	vehicle (acute)									*

403	WT	DHF	*								
404	WT	DHF	*								
405	WT	DHF	*								
406	WT	DHF	*								
407	WT	DHF	*								
408	WT	DHF	*		*						
409	WT	DHF	*		*						
410	WT	DHF	*		*						
411	WT	DHF	*								
412	WT	DHF	*		*						
413	WT	DHF	*		*						
414	WT	DHF	*		*						
415	WT	DHF	*		*						
416	WT	DHF	*		*						
417	WT	DHF	*		*						
418	WT	DHF	*								
419	WT	DHF	*								
420	WT	DHF	*								
421	WT	DHF			*						
422	WT	DHF		*							
423	WT	DHF		*							
424	WT	DHF		*							
425	WT	DHF		*							
426	WT	DHF		*							
427	WT	DHF		*							
428	WT	DHF		*							
429	WT	DHF		*							
430	WT	DHF		*							
431	WT	DHF		*							
432	WT	DHF		*							
433	WT	DHF		*							
434	WT	DHF		*							
435	WT	DHF		*							
436	WT	DHF		*							
437	WT	DHF		*							
438	WT	DHF		*							
439	WT	DHF		*							
440	WT	DHF								*	
441	WT	DHF								*	
442	WT	DHF								*	
443	WT	DHF								*	
444	WT	DHF									*
445	WT	DHF									*
446	WT	DHF									*
447	WT	DHF									*
448	WT	DHF									*
449	WT	DHF									*
450	WT	DHF									*
451	WT	DHF									*

452	WT	DHF									*
453	WT	DHF									*
454	WT	DHF									*
455	WT	DHF									*
456	WT	DHF (acute)									*
457	WT	DHF (acute)									*
458	WT	DHF (acute)									*
459	WT	DHF (acute)									*
460	WT	DHF (acute)									*
461	WT	DHF (acute)									*
462	WT	DHF (acute)									*
463	WT	DHF (acute)									*
464	WT	DHF (acute)									*
465	WT	DHF (acute)									*
466	WT	DHF (acute)									*
467	WT	DHF (acute)									*
468	WT	DHF (acute)									*
469	WT	DHF (acute)									*
470	WT	DHF (acute)									*
471	WT	DHF (acute)									*
472	WT	DHF (acute)									*
473	WT	DHF (acute)									*
474	WT	DHF (acute)									*
475	WT	DHF (acute)									*
476	Ts65Dn	DHF	*								
477	Ts65Dn	DHF	*								
478	Ts65Dn	DHF	*								
479	Ts65Dn	DHF	*								
480	Ts65Dn	DHF	*								
481	Ts65Dn	DHF	*								
482	Ts65Dn	DHF	*								
483	Ts65Dn	DHF	*								
484	Ts65Dn	DHF	*		*						
485	Ts65Dn	DHF	*		*						
486	Ts65Dn	DHF	*		*						
487	Ts65Dn	DHF	*		*						
488	Ts65Dn	DHF	*		*						
489	Ts65Dn	DHF	*		*						
490	Ts65Dn	DHF	*		*						
491	Ts65Dn	DHF	*								
492	Ts65Dn	DHF	*								
493	Ts65Dn	DHF	*								
494	Ts65Dn	DHF		*							
495	Ts65Dn	DHF		*							
496	Ts65Dn	DHF		*							
497	Ts65Dn	DHF		*							
498	Ts65Dn	DHF		*							
499	Ts65Dn	DHF		*							
500	Ts65Dn	DHF		*							

501	Ts65Dn	DHF		*							
502	Ts65Dn	DHF		*							
503	Ts65Dn	DHF		*							
504	Ts65Dn	DHF		*							
505	Ts65Dn	DHF								*	
506	Ts65Dn	DHF								*	
507	Ts65Dn	DHF								*	
508	Ts65Dn	DHF									*
509	Ts65Dn	DHF									*
510	Ts65Dn	DHF									*
511	Ts65Dn	DHF									*
512	Ts65Dn	DHF									*
513	Ts65Dn	DHF									*
514	Ts65Dn	DHF									*
515	Ts65Dn	DHF									*
516	Ts65Dn	DHF									*
517	Ts65Dn	DHF									*
518	Ts65Dn	DHF									*
519	Ts65Dn	DHF									*
520	Ts65Dn	DHF (acute)									*
521	Ts65Dn	DHF (acute)									*
522	Ts65Dn	DHF (acute)									*
523	Ts65Dn	DHF (acute)									*
524	Ts65Dn	DHF (acute)									*
525	Ts65Dn	DHF (acute)									*
526	Ts65Dn	DHF (acute)									*
527	Ts65Dn	DHF (acute)									*
528	Ts65Dn	DHF (acute)									*
529	Ts65Dn	DHF (acute)									*
530	Ts65Dn	DHF (acute)									*
531	Ts65Dn	DHF (acute)									*
532	Ts65Dn	DHF (acute)									*
533	Ts65Dn	DHF (acute)									*
534	Ts65Dn	DHF (acute)									*
535	Ts65Dn	DHF (acute)									*
536	Ts65Dn	DHF (acute)									*
537	Ts65Dn	DHF (acute)									*
538	Ts65Dn	DHF (acute)									*
539	Ts65Dn	DHF (acute)									*

Asterisk indicates the experimental procedure/test carried out on each mouse.
NOR: Novel Object Recognition test. OL: Object Location test. CFC: Contextual Fear Conditioning test. IHC: Immunohistochemistry. EM: Electron Microscopy. EPhy: Electrophysiology. Bioch: Biochemistry.
Mice highlighted in yellow did perform the NOR or OL behavioral tests, but could not be included in the study because we lost the video-recording files of the behavioral tests due to the failure of a hard-drive in which they were stored. Files could not be recovered.

Supplementary references

Costa, A. C. S., M. R. Stasko, C. Schmidt and M. T. Davisson (2010). "Behavioral validation of the Ts65Dn mouse model for Down syndrome of a genetic background free of the retinal degeneration mutation Pde6brd1." Behavioural Brain Research **206**: 52-62.
